# Development of a TLR7/8 agonist adjuvant formulation to overcome early life hyporesponsiveness to DTaP vaccination

**DOI:** 10.1038/s41598-022-20346-w

**Published:** 2022-10-18

**Authors:** David J. Dowling, Soumik Barman, Alyson J. Smith, Francesco Borriello, Danielle Chaney, Spencer E. Brightman, Gandolina Melhem, Byron Brook, Manisha Menon, Dheeraj Soni, Simone Schüller, Karthik Siram, Etsuro Nanishi, Hélène G. Bazin, David J. Burkhart, Ofer Levy, Jay T. Evans

**Affiliations:** 1 Precision Vaccines Program, Division of Infectious Diseases, Boston Children’s Hospital, Harvard Institutes of Medicine, Room 842, 4 Blackfan Circle, Boston, MA 02115 USA; 2 Harvard Medical School, Boston, MA USA; 3 Center for Translational Medicine, University of Montana, 32 Campus Drive, Missoula, MT 59802 USA; 4 Department of Translational Medical Sciences, Center for Basic and Clinical Immunology Research (CISI), University of Naples Federico II, Naples, 80131 Italy; 5 WAO Center of Excellence, Naples, 80131 Italy; 6 Department of Biomedical and Pharmaceutical Sciences, University of Montana, Missoula, MT USA; 7 Broad Institute of MIT & Harvard, Cambridge, MA USA; 8 Present Address: Seagen, Bothell, WA USA; 9 Present Address: Generate Biomedicines, Cambridge, MA USA

**Keywords:** Adjuvants, Toll-like receptors

## Abstract

Infection is the most common cause of mortality early in life, yet the broad potential of immunization is not fully realized in this vulnerable population. Most vaccines are administered during infancy and childhood, but in some cases the full benefit of vaccination is not realized in-part. New adjuvants are cardinal to further optimize current immunization approaches for early life. However, only a few classes of adjuvants are presently incorporated in vaccines approved for human use. Recent advances in the discovery and delivery of Toll-like receptor (TLR) agonist adjuvants have provided a new toolbox for vaccinologists. Prominent among these candidate adjuvants are synthetic small molecule TLR7/8 agonists. The development of an effective infant *Bordetella pertussis* vaccine is urgently required because of the resurgence of pertussis in many countries, contemporaneous to the switch from whole cell to acellular vaccines. In this context, TLR7/8 adjuvant based vaccine formulation strategies may be a promising tool to enhance and accelerate early life immunity by acellular *B. pertussis* vaccines. In the present study, we optimized (a) the formulation delivery system, (b) structure, and (c) immunologic activity of novel small molecule imidazoquinoline TLR7/8 adjuvants towards human infant leukocytes, including dendritic cells. Upon immunization of neonatal mice, this TLR7/8 adjuvant overcame neonatal hyporesponsiveness to acellular pertussis vaccination by driving a T helper (Th)1/Th17 biased T cell- and IgG2c-skewed humoral response to a licensed acellular vaccine (DTaP). This potent immunization strategy may represent a new paradigm for effective immunization against pertussis and other pathogens in early life.

## Introduction

Whooping cough is a respiratory infection caused by the Gram-negative encapsulated bacterium *Bordetella pertussis*, which can lead to severe complications and death, especially in newborns and young infants^1,2^. Vaccination remains the most effective strategy to prevent whooping cough. According to the World Health Organization, worldwide vaccination programs started in the mid-twentieth century reduced the incidence of *B. pertussis* infection and associated mortality by 90%^3^. Currently employed acellular pertussis (aP) vaccines significantly enhance protection against *B. pertussis* but require 5 doses starting from 2 months through 4–6 years of age and immunity can wane over time^4,5^. As such, there remains a need for booster immunizations every 10 years in adults which results in low vaccination rates in this group, including pregnant women with inadequate anti-*B. pertussis* IgG^6–8^, further contributing to suboptimal protection in neonates and infants. Notably, since 2010, up to 50,000 cases of whooping cough have been reported each year in the United States, interspersed with prominent epidemics^9^.

Given the global burden of pertussis infection, an improved infant *B. pertussis* vaccine is now urgently needed^10^. Although the majority of global immunization is pediatric, development of new *B. pertussis* vaccines has typically taken an ad hoc approach that disregards age-specific immunity in preclinical discovery and development, with an initial focus on adult vaccine trials^11^. Limited vaccine immunogenicity and transient presence of neutralizing antibodies (Abs) are major challenges when immunizing newborns and young infants^12^. Historically, solutions to these challenges included increasing immunogenicity via use of multi-dose pediatric immunization schedules, expanding the length of time between vaccine doses and/or administration of doses later in infancy^13^, or increasing/modifying antigen-content^14^. While these approaches enhanced immunogenicity, they require multiple doses and waning immunity remains a challenge.

Novel adjuvant strategies tailored to enhance early life immunity represent an innovative approach to addressing limited vaccine immunogenicity in the very young^15–18^. Accordingly, the development of rationally designed vaccine formulations, which include adjuvants that more effectively enhance immune responses in childhood, is a promising approach^19–21^. In particular, there is an emerging interest in agonists of intracellular TLR7 and TLR8, endosomal pattern recognition receptors (PRRs) whose natural ligands are microbial RNA^22^. There may be benefit to dual activation of these pathways as TLR7 stimulation activates B cell proliferation, IgG production and class switching^23^, while TLR8 is a vita-PAMP (viability associated-pathogen associated molecular patterns) receptor and facilitates T follicular helper (T_FH_) cell differentiation by IL-12 signal cascade and is a promising target for T_FH_ cell-skewing vaccine adjuvants^22^. Of note, TLR7/8 agonists (TLR7/8As) are refractory to inhibitory plasma adenosine pathways in newborns^21,24^ and robustly activate the early life immune system in vitro and in vivo. Unlike agonists of most TLRs, that fail to elicit Th1 cytokine production from human newborn and infant leukocytes, TLR7/8-activating imidazoquinoline compounds (IMQs) induce robust Th1-polarizing responses in both human neonatal and adult dendritic cells (DCs)^21,25,26^. TLR7/8 stimulation can also induce robust B cell responses in early life^27–29^. Addition of a TLR7A to aP vaccine in vivo enhanced adult murine Th1 and Th17 responses and protective immunity^30^, but corresponding data in infant animals to our knowledge are lacking.

Formulation science has traditionally been underappreciated in adjuvant discovery and development^31^. However over the past decade, with growing realization of the importance of formulation, the ability to develop more fully characterized vaccine formulations has become a major goal for many involved in the areas of vaccine development^32^. Formulation science has played a significant role in facilitating the delivery of TLR7/8As as vaccine adjuvants^33^. Synthetic IMQ TLR7/8A small molecules formulated in an aqueous dispersion have limited adjuvant activity since they are prone to diffuse away from the injection site, which can also result in systemic cytokine induction and toxicity^34^. Most notably, R848 has a poor tolerability profile when tested systemically in humans. Common systemic side effects of this aqueous formulation include injection site reactogenicity and flu-like symptoms (fever, headache and malaise) correlating to systemic immune activation, seen with high peripheral blood concentrations of numerous inflammatory cytokines^35,36^. We have previously demonstrated that TLR7/8 adjuvantation can overcome newborn hyporesponsiveness to pneumococcal conjugate vaccination at birth^37^. 3M-052, a locally-acting lipidated IMQ TLR7/8A adjuvant bearing a fatty acid tail (C18 lipid moiety), drove robust Th1 cytokine production by human newborn leukocytes in vitro, both alone and in synergy with the alum-adjuvanted pneumococcal conjugate vaccine (PCV)13^37^. Moreover, a single administration of 3M-052 in combination with PCV13 on the first day of life, accelerated and enhanced anti-*S. pneumoniae* polysaccharide-specific B cells, serotype-specific Ab titers, and Ab-mediated phagocytic killing, to serum concentrations ~ 10–100 times greater than a single birth dose of PCV13 alone. A very recent study also demonstrated the efficacy of 3M-052 in induction of HIV-1 envelope specific long-lived plasma cells and humoral immunity in non-human primates^38^. These studies suggested that appropriately formulated TLR7/8A adjuvants, potentially co-administered with alum, can enhance responses to vaccines in newborns and young infants^37^.

In this study, we demonstrate that immunization of newborn mice with TLR7/8A:alum- adjuvanted DTaP vaccine drives an antigen specific Th1/Th17-polarized T cell response along with a balanced humoral response characterized by enhanced switch toward an IgG2a/c subclass. Rational design of precision adjuvants at the preclinical discovery and development phase may thus identify adjuvanted vaccine formulations that optimally shield vulnerable populations^17^. Such an approach may be broadly applicable, potentially closing the window of vulnerability to infections in early life.

## Results

### TLR7/8 agonist UM-3001 demonstrates robust induction of Th1- and Th2-polarizing cytokines in human newborn cord blood

**Figure 1:**
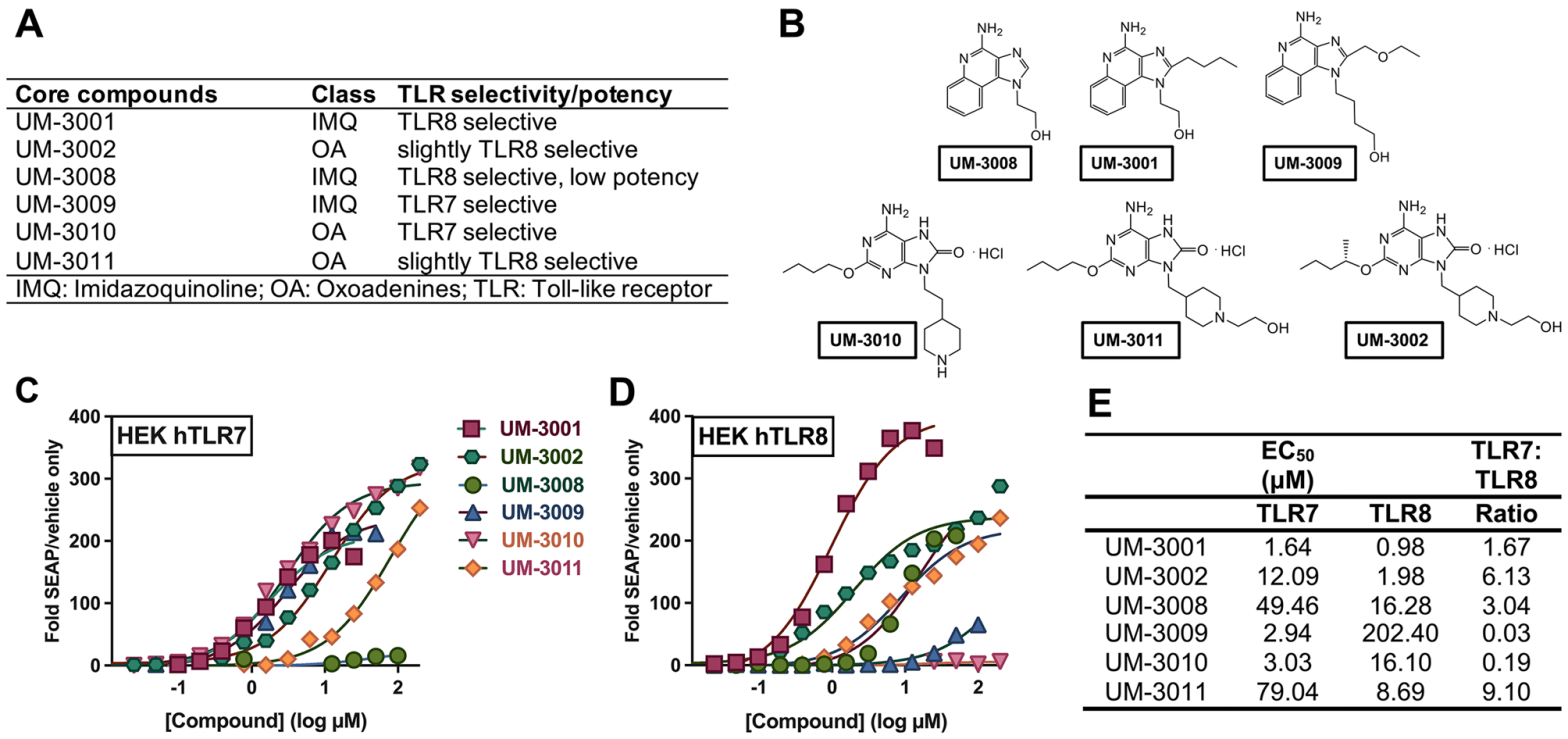
Overview of the core-aqueous imidazoquinoline and oxoadenine scaffolds. (**A**) Naming convention, chemical class and TLR selectively of each core scaffolds^43,60,75,76^; (**B**) core imidazoquinolines and core oxoadenines investigated in this study. (**C**–**D**) Six TLR agonists were compared. HEK-293 cells transfected with (**C**) human TLR7 or (**D**) human TLR8 and an NF-kB-driven reporter SEAP gene were stimulated for 18–24 h with TLR agonists. The y-axis shows the level of SEAP activity as a fold change over unstimulated cells. The x-axis shows the concentration of each compound in μM (log_10_ scale). Each data point represents the mean of triplicate culture wells, and representative of three separate experiments. (**E**) Amongst the IMQ and OA compounds evaluated in a HEK293 assay, UM-3001 was the most potent with lowest half maximal effective concentration (EC_50_) for both TLR7 and TLR8, with a preference for TLR8.

The use of novel TLR7/8As as adjuvants has been pursued in recent years as an effective method to increase immune responsiveness to several different vaccines and antigens^33,39^. To determine if these compounds are also able to adjuvant the immune response to the aP vaccine, six novel imidazoquinoline (IMQ) and oxoadenine (OA) scaffold-based compounds were synthesized as described previously^40–42^ and evaluated^43^. Three IMQ compounds, UM-3001, UM-3008 and UM-3009 (Fig. 1A,B) and three OA compounds, UM-3002, UM-3010 and UM-3011 (Fig. 1A,B), had variable TLR7 and TLR8 selectivity and potency in HEK293 assay (Fig. 1C,D). The precise structure–activity-relationship (SAR) leading to the TLR7 and TLR8 selectivity and potency of these TLR7/8As has been reported previously^39,41,42^. UM-3001 had the greatest potency for both hTLR7 and hTLR8 (Fig. 1C–E).

**Figure 2:**
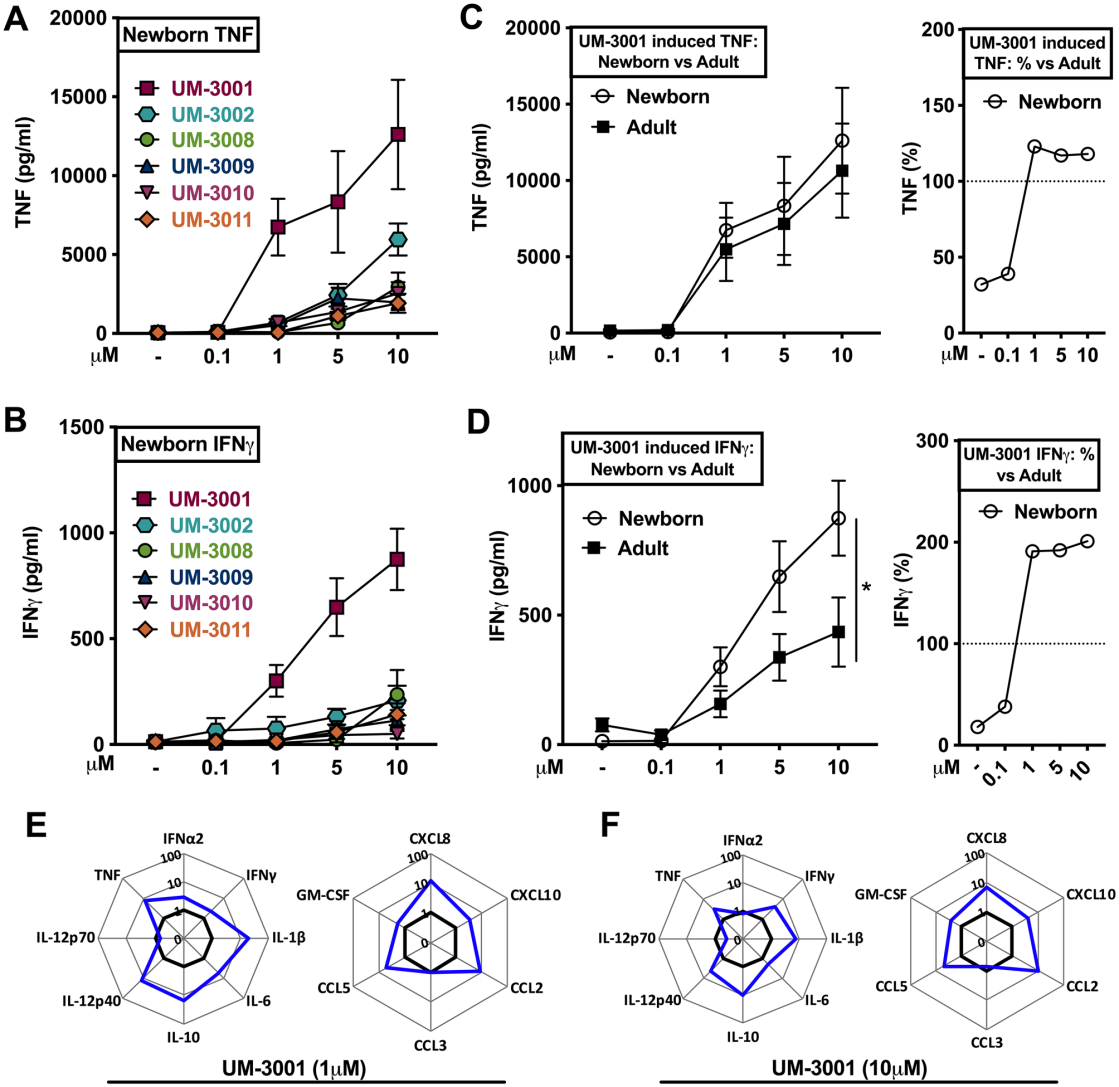
Imidazoquinoline UM-3001 demonstrated age-specific potency, effectiveness and IFNγ production in human newborn cord blood. (**A**–**B**) Human neonatal cord blood cultured in vitro for 6 h with buffer control (RPMI) or with increasing concentrations of various UM adjuvants. (**C**–**D**) Newborn versus adult blood cultured in vitro for 6 h with UM-3001. Supernatants were collected for ELISA. Results represent mean ± SEM, A-B; n = 7, C-D; n = 7. Cell supernatants were analyzed for cytokine expression by multiplex assay. Data are shown as fold change for newborn cord (blue line) over adult stimulated whole blood (black line) for 1 μM (**E**) and 10 μM (**F**) of UM-3001. (n = 6 adults, n = 8 newborns). For comparisons between overall groups (e.g., newborn vs. adult), two-way ANOVA followed by Tukey’s test for multiple comparisons was applied, and statistical significance denoted as *p < 0.033.

We next tested the ability of the core IMQs and OAs to induce concentration-dependent cytokine production in in vitro human neonatal and adult whole blood assays (Fig. 2A–D). While the vehicle control did not induce TNF or IFNγ production, all six of the TLR7/8As tested demonstrated titration-dependent induction of TNF (Fig. 2A) and IFNγ (Fig. 2B) from newborn cord blood. Interestingly, UM-3001 demonstrated adult-like potency and effectiveness with respect to induction of TNF production in newborn cord blood (Fig. 2C). Of note, UM-3001 demonstrated the greatest potency, effectiveness and IFNγ polarization in newborn cord blood. UM-3001-induced IFNγ production in newborn cord blood was most evident at 10 μM, reaching ~ 900 pg/ml, twice the IFNγ concentration produced in similarly stimulated adult blood (Fig. 2D, *p* < 0.033).

UM-3001 also demonstrated a broader ability to induce a newborn-specific cytokine and chemokine potency and polarization. Supernatants derived from stimulated whole blood assays were analyzed for cytokine/chemokine/interferon expression by multiplex assay. Results were graphed as fold change for newborn cord blood (blue line) over adult (black line), at both a low (1 μM, Fig. 2E, Table S1) and high (10 μM, Fig. 2F, Table S1) concentration of UM-3001 which demonstrated concentration-dependent robust accumulation of all cytokines tested including IL-1β, IL-6 and IL-10 (Fig. 2E,F , Table S1). UM-3001 also induced significant production of chemokines including CXCL8 and CCL2 as well as GM-CSF in stimulated human newborn cord- vs adult blood (Fig. 2E,F, Table S1).

Next, we employed a human newborn Th1 polarization assay, which leveraged the intrinsic characteristics of the newborn T cell compartment, composed mainly of naïve T cells, to evaluate how UM-3001-induced T cell polarization in a mixed mononuclear cell culture in the presence of a T cell receptor (TCR)-mediated stimulus (Fig. S1A). Additionally, cord blood mononuclear cells (CBMCs) were cultured in the presence of autologous plasma, a rich source of age-specific soluble immunomodulatory factors to closer mimic in vivo conditions^44^. Neonatal CBMCs stimulated for 96 h (hrs) with αCD3 (polyclonal T cell activator) and 10 μM of UM-3001 demonstrated significantly enhanced production of IFNγ by newborn T cells (Fig. S1B, *p* < 0.033).

### Targeted lipidation changes the immunostimulatory properties of the IMQ scaffold

**Figure 3:**
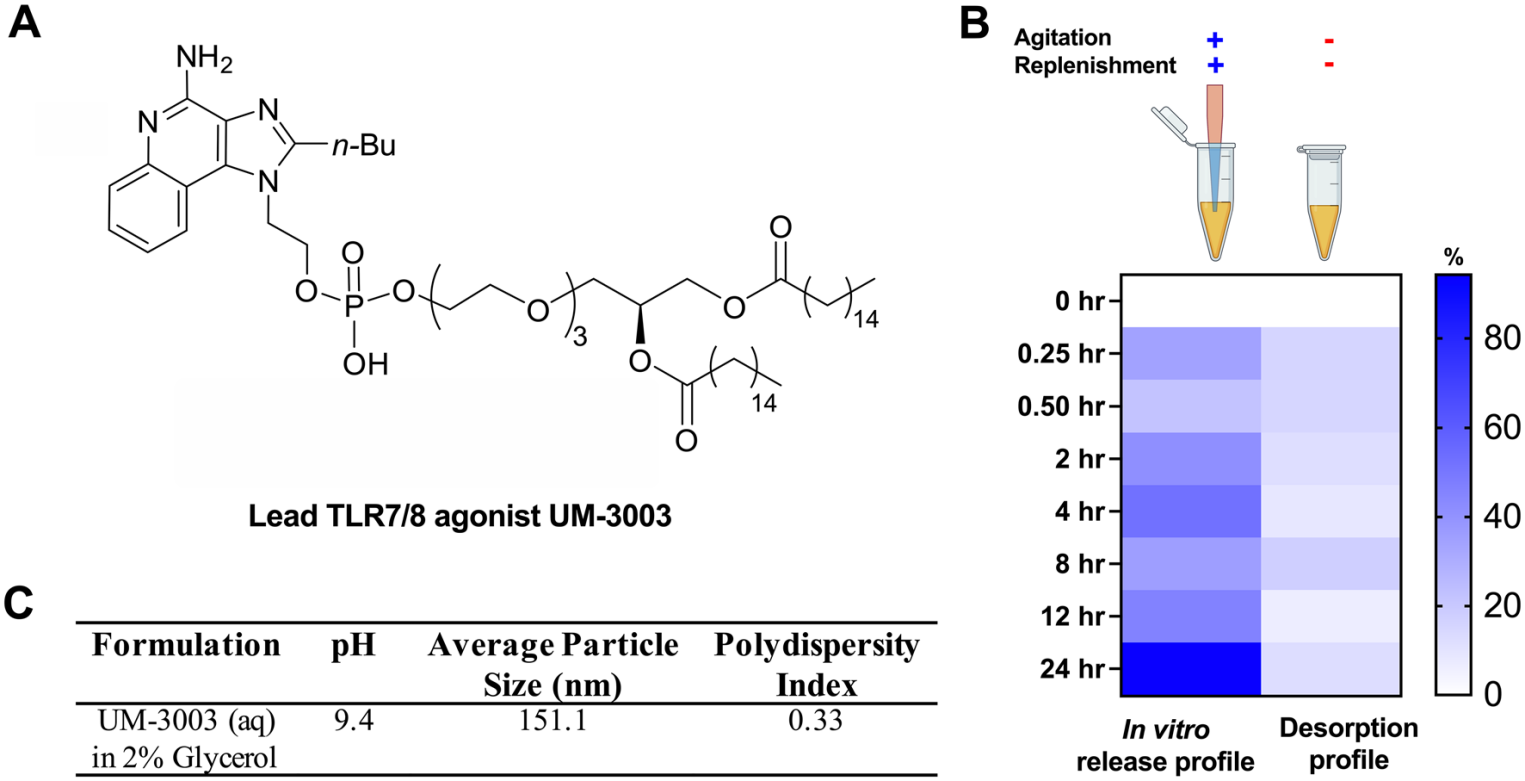
The optimized phospholipidated TLR7/8 agonist UM-3003 rapidly and fully adsorbed onto alum. (**A**) Chemical structure of the lead TLR7/8 agonist UM-3003. The phosphorylated derivative of the IMQ UM-3001 was modified through introduction of a polyethylene glycol (PEG) linker of 3 repeating units generating a compound that was named UM-3003. (**B**) In vitro release and desorption profile of UM-3003 from UM-3003 alhydrogel adsorbed formulation in adjuvant free human plasma. Percentage desorption is distributed to scale from 0 to 100. (**C**) Average particle size, pH and polydispersity index of UM-3003 in 2% glycerol.

Upon determining that these agonists, and UM-3001 in particular, significantly enhance newborn TNF and IFNγ responses, we further explored their utility as adjuvants upon derivatization using lipid conjugation. Locally acting adjuvants may have distinct advantages in vivo by avoiding extensive systemic distribution and consequent induction of systemic inflammation^45–47^. In addition, cellular uptake is a prerequisite for cellular activation in response to TLR7/8As since these receptors are localized in the endosomal/lysosomal compartments^48^. Accordingly, there is interest in strategies to increase the penetration of the TLR7/8As into the endosome of DCs and other immune cells which, in addition to targeting potent response, may also ameliorate potentially reactogenic or low-level toxic side-effects. In this context, lipid conjugation of nucleoside drugs including TLR7/8As^49^ can facilitate endocytosis, enhance oral bioavailability, and decrease systemic side effects by creating a depot effect. The basic IMQ pharmacophore was optimized to contain a 2 *n*-butyl and 1 ethanol groups creating UM-3001 (Fig. 1B). For nucleolipidation studies this core was further derivatized by phospholipidation^50^ of the ethanol at the 1 position, followed by addition of an optimally determined 3 polyethylene glycol (PEG) linker^51^ (Fig. 3A). Of note, UM-3001 core compound does not contain a phosphate moiety and thus cannot adsorb to alum (Table S2), a common vaccine component serving as an adjuvant and antigen stabilizer and addition of a phosphate or phospholipid on the core can facilitate alum adsorption. Addition of the PEG linker between the phosphate and lipid moiety, to UM-3001 substantially changed the agonist’s cytokine skewing properties from pro-inflammatory biased, TNF production, to type I interferon-biased (unpublished observations). Of note, while potency and efficacy with respect to TNF induction was directly correlated with the number of PEG units, for IFNα, the SAR was less clear, although the > 3 PEG linker compound displayed greater efficacy and potency for induction of this cytokine (unpublished observations). This change in phenotype informed selection of a compound with 3 PEG units (hereby UM-3003), that displayed a skewed type I interferon response with minimal inflammatory cytokine production suggesting it would have reduced reactogenicity while maintaining immunogenicity.

### Lipidated TLR7/8 agonist rapidly and fully adsorbed onto alum

Collectively, these observations support the potential of both the core and lipidated molecules to serve as immunomodulatory vaccine adjuvants. To investigate the ability of these novel TLR7/8As to augment response to aP vaccine, we characterized their ability to enhance immunogenicity of a commercially utilized, Food and Drug Administration (FDA) approved, pediatric DTaP vaccine (Trade name: *Infanrix*). The acellular vaccine contains three recombinant *B. pertussis* proteins, the inactivated pertussis toxin (PT), the adhesin filamentous hemagglutinin (FHA), and outer membrane protein, pertactin (PRN). All three proteins are unstable unless adsorbed to aluminum hydroxide (alum). The nucleolipid derivative compound(s) contain phosphate groups which should theoretically adsorb to alum in the aP vaccine. Accordingly, we undertook an adsorption study to determine how the different IMQ adjuvant compounds would interact with the final vaccine antigen formulation (DTaP). Aqueous suspensions of the lipidated compound UM-3003 and its parent pharmacophore UM-3001 were incubated with DTaP vaccine and aliquots were assayed at various time points (1, 2, 24 h) post admixture to assess amounts of unbound compounds in the supernatant via reversed-phase high-performance liquid chromatography (RP-HPLC). As suspected, the lipidated compound fully adsorbed (~ 96–100%) to the alum/antigen within 1 h (Table 1). The addition of excess alum, through pre-adsorption of the compound on the alum, had little effect on UM-3003 adsorption (~ 99% after 1 h) (Table 1, top panel). However, under all conditions tested, the core UM-3001 compound demonstrated very limited ability to adsorb to aP vaccine- i.e., ~ 4–7% within 1–2 h (Table S2), with peak area intensity levels similar to the unmixed controls. This was further addressed by investigating adsorption strength and the desorption profile of UM-3003 from alum.

**Table 1:** Phospholipidation enables adsorption of UM-3003 to alum and *Infanrix* vaccine.

Sample (Alum: UM-3003 Salt)	Time point	Time (min)	Peak area	% Adsorbed
1:2 Alum: UM-3003 (aq)	0 (< 5 min)	12	19.81	96.5
	15 min	18	8.41	98.5
	30 min	34	0.06	100.0
	60 min	68	n.a	100.0
	23 h	1380	n.a	100.0
1:1 Alum: UM-3003 (aq)	0 (< 5 min)	12	12.04	97.8
	15 min	18	n.a	100.0
	30 min	34	n.a	100.0
	60 min	68	n.a	100.0
	23 h	1380	n.a	100.0
Alum control	0 (< 5 min)		n.a	0.0
	23 h		n.a	0.0
UM-3003 (aq) control	0 (< 5 min)		160.1	
	23 h		175.11	
1:4 *Infanrix* Alum: UM-3003 (aq)		1		99.1
		5		99.4
		7		99.9
		10		99.9
		15		100.0
		30		100.0
		76		100.0
		180		99.9
		1260		99.9

Adsorption kinetics of UM-3003 [molecular weight (MW) of 1047.42)] onto 2% alhydrogel alum and DTaP alum respectively. Aluminum hydroxide derived from DTaP (*Infanrix*) vaccine, which has preexisting pertussis antigen adsorbed to its surface, was evaluated up to 23 h. UM-3003 fully adsorbed (~ 96–100%) to the alum/antigen within 30 min, with or without excess alum.

### Predicable in vitro release and desorption kinetics of lipidated TLR7/8 agonist from alum

The presence of various ions in plasma including calcium, potassium, sodium, magnesium, phosphate, chloride, bicarbonate, etc., can facilitate desorption of the adsorbed compounds from the surface of alhydrogel by ligand exchange^52^. To evaluate the adsorption strength and desorption profile of UM-3003 from its adsorbed form on alhydrogel, we performed in vitro release and desorption studies upon exposure to plasma over a period of 24 h. The in vitro release and desorption profiles of UM-3003 from UM-3003 adsorbed alhydrogel formulations are shown in Fig. 3B. Without agitation and periodical replenishment of the release medium, ~ 18% of UM-3003 was desorbed from a UM-3003-adsorbed alhydrogel formulation, suggesting strong adsorption of UM-3003 onto alhydrogel. The amount of UM-3003 desorbed from alhydrogel increased to ~ 90% with agitation and periodical replenishment of the release medium with fresh adjuvant-free human plasma, suggesting enhanced desorption. Periodical replenishment with fresh adjuvant-free human plasma likely provided additional ions to desorb UM-3003 from the surface of alhydrogel by ligand exchange. To identify the most likely mechanism of release of UM-3003 from UM-3003 adsorbed alhydrogel formulation, the release profile was fitted with various kinetic models such as zero order (R^2^ = 0.73), first order (R^2^ = 0.51), Higuchi, Korsmeyer-Peppas (R^2^ = 0.7540; n = 0.283) and Hixson-Crowell (0.36). The results indicated that Korsmeyer-Peppas model was suitable to explain the mechanism of release of UM-3003 from alhydrogel. A corresponding n value of 0.283 in this model indicated that UM-3003 desorbed from alhydrogel via Fickian diffusion i.e., the release of UM-3003 in the release medium was from a region of higher concentration to lower concentration^53^. Lastly, UM-3003 in 2% glycerol presented with an average particle size of ~ 151 nm, a pH of ~ 9.4 and a polydispersity index of 0.33 (Fig. 3C). This aqueous formulation of UM-3003 is suitable for sterile filtration due to its low particle size and facilitates facile adsorption to alhydrogel by direct addition.

### Lipid adsorption to alum unlocks TLR7/8 adjuvanticity

Formulations of DTaP:TLR7/8A were next tested for immunogenicity and dosing in adult BALB/c and C57BL/6 mice. First, BALB/c mice were immunized twice, 14 days apart with *Infanrix* (1/100th of the human dose) ± UM-3001 or UM-3003 at 0.1, 1 or 10 µg per mouse in different formulations. Serum was harvested 14 days following prime and boost (Fig. S2A). At two weeks post primary vaccination, anti-FHA IgG2a serum Ab titers were significantly elevated (~ sixteenfold) over *Infanrix* alone vaccinated mice when 10 µg of UM-3003, with (*p* < 0.001) or without alum (*p* < 0.001) pre-adsorption of the adjuvant, was included in the vaccine (Fig. S2C). IgG1 anti-FHA titers were only significantly boosted (~ fourfold) with UM-3003 pre-adsorbed on alum (*p* < 0.001) (Fig. S2B) compared to *Infanrix* alone vaccinated mice demonstrating a potential difference in effect for alum adsorption in driving a balanced Th1/Th2 immune response or a preferred Th1 response. By 14 days post-secondary immunization serum anti-FHA titers were much higher than *Infanrix* alone and nearing a plateau for many groups (Fig. S2D, E). Again, we noted that addition of UM-3003 both with and without pre-alum adsorption facilitated significantly higher IgG1 and IgG2a Ab titers. For both 14 days post-primary and -secondary immunization of adult mice, addition of the core IMQ UM-3001 did not enhance either IgG1 or IgG2a Ab titers. Of note, increase of IgG2a Ab titers during secondary immunization were observed when boosting with a 10 μg dose of UM-3003 alone (*p* < 0.001) and a 0.1 μg dose of UM-3003 pre-adsorbed on alum (Fig. S2E*, p* < 0.002).

**Figure 4:**
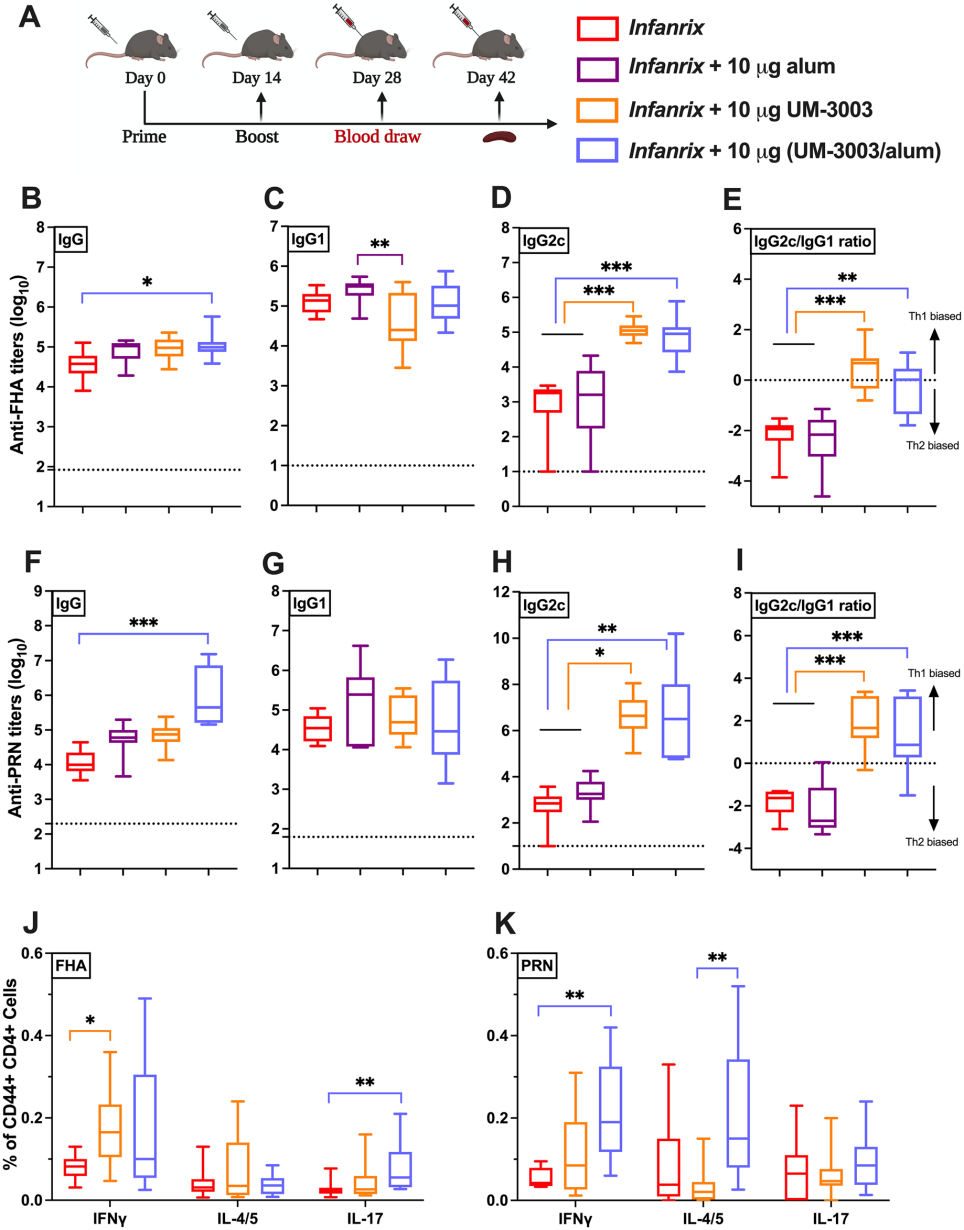
UM-3003 enhances DTaP specific IgG2c production and Th1 polarization in adult mice. Adult C57BL/6 mice were immunized twice, 14 days apart (**A**) with DTaP (1/100th of the human dose) ± UM-3003 or alum at 10 µg per mouse in different formulations (aqueous or pre-adsorbed to alum). Serum was harvested 14 days following boost (**B**–**I**). Anti-FHA (**B**-**E**) and anti-PRN (**F**–**I**) serum Ab IgG (**B**, **F**), IgG1 (**C**, **G**) and IgG2c (**D**, **H**) titers were measured by ELISA. PBS control groups were represented as dotted line. (**E**, **I**) IgG2c/IgG1 ratios for FHA and PRN respectively. (**J**, **K**) Murine CD4^+^ T cell responses after FHA and PRN stimulation. Four weeks following booster, splenocytes from DTaP and adjuvanted vaccinates were isolated, stimulated with 2 μg/ml of either FHA or PRN along with CD28 (1 μg/ml) and CD49d (1 μg/ml) for 12 h followed by 6 h of BFA stimulation to block the extracellular cytokine secretion. After stimulation, cells were harvested, stained (intracellular cytokine staining) and analyzed by flow cytometry. Plots were gated on CD44^+^ CD4^+^ lymphocytes and analyzed for all combinations of simultaneous IFNγ, IL-4/5 and IL-17A productivity. Statistical analysis was performed by nonparametric Kruskal–Wallis test corrected for multiple comparisons; *p < 0.033, **p < 0.002, ***p < 0.001 (n = 8–15 per group). Study is inclusive of three independent repeats.

Next, DTaP:TLR7/8A induced immunogenicity in adult C57BL/6 mice was confirmed (Fig. 4A-I), with the IgG2c induced phenotype mirroring those observed in BALB/c mice. At two weeks post-secondary vaccination of DTaP (1/100th of human dose) with 10 µg of UM-3003, anti-FHA and anti-PRN IgG2c serum Ab titers were significantly elevated over DTaP alone (*p* < 0.001 and *p* < 0.033, respectively) or with additional alum (10 µg, *p* < 0.001 and *p* < 0.002, respectively) (Fig. 4D,H). A higher IgG2c/IgG1 ratio (around or over 1) was observed in UM-3003 adjuvanted groups suggesting Th1 biased Ab response (Fig. 4E,I). Via evaluation of FHA- and PRN-specific memory T cell responses post vaccination, we also confirmed intracellular IFNγ production by splenic effector CD44^+^ CD4^+^ T cells (which denotes Th1 skewed response) from DTaP:TLR7/8A immunized mice (Fig. 4J,K and S3) following antigen (both FHA and PRN) stimulation. Additional alum adjuvantation alone did not play any significant role to stimulate DTaP experienced T cells (unpublished observations). A similar trend was observed when IFNγ secretion by splenocytes, measured by multiplex assay from adult immunized mice following stimulation for 72–80 h (Fig. S4A,D), where both DTaP:TLR7/8A formulations enhanced antigen specific Th1-cytokine shifts compared to DTaP alone.

### TLR7/8 stimulation of neonatal MoDCs elicits a mixed Th polarizing innate cytokine response

**Figure 5:**
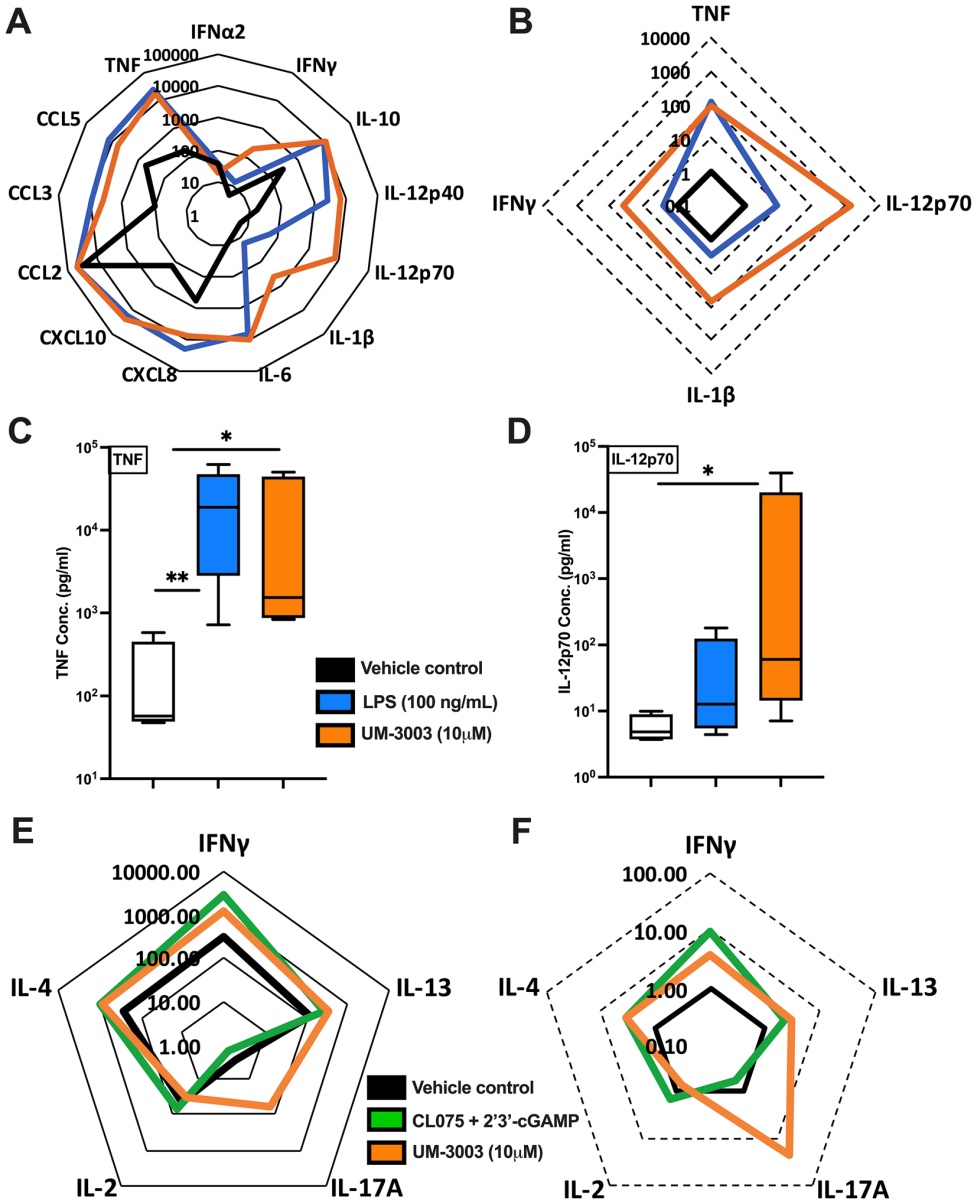
Lipidated imidazoquinoline UM-3003 demonstrates robust potency and efficacy in inducing Th-polarizing cytokines from human newborn MoDCs. (**A**–**B**) Newborn MoDCs were stimulated for 24 h and supernatants were collected for multiplex assay. Cytokine responses (pg/ml) of 13 analytes (n = 5) were represented in radar plot (**A**) demonstrating neonatal MoDCs concurrently stimulated in the presence of 2% glycerin (vehicle control represented by thick black line) or 100 ng/ml of LPS (blue line) or 10 μM of UM-3003 (orange line). (**B**) Fold-change of 100 ng/ml of LPS (blue line) or 10 μM of UM-3003 (orange line) over vehicle control (thick black line) are shown. (**C**) TNF (n = 5), (**D**) IL-12p70 (n = 5) ELISA were performed by stimulated supernatants where lipidated compound demonstrating similar TNF induction ability compared with LPS, but slightly increased IL-12p70 production. In, (**E**–**F**), cord blood mononuclear cells (CBMCs) (n = 6) were stimulated in the presence of the polyclonal T cell activator α-CD3 (5 μg/ml) in combination or alone for 96 h. TLR7/8 agonist (CL075, 10 μM) and STING agonist (2′3′-cGAMP, 10 μM) in combination were used as a benchmark control. (**E**) Radar plot demonstrated total cytokine levels (pg/ml) in cell free supernatants by a high sensitivity T cell multiplex assay from six independent experiments. (**F**) Fold changes relative to vehicle control are shown. Statistical analysis was performed by nonparametric Kruskal–Wallis test corrected for multiple comparisons and statistical significance denoted as *p < 0.033, **p < 0.002.

As antigen presenting cells are the key target for candidate adjuvants, we compared concentration-dependent responses (0.1 μM to 10 μM) of UM-3001 and UM-3003 in vitro with human adult and neonatal MoDCs (Fig. 5A–D, Table S3A,B, Fig. S5). Cytokine-inducing activity of UM-3003 was also tested in newborn mononuclear cell culture in the presence of α-CD3 which triggers TCR stimulation (Fig. 5E,F, Table S3C,D). This approach was employed to assess the impact of formulation and/or lipidation to modulate the ability of a TLR7/8A to activate MoDCs and/or polarize T cells. Both the TLR4A LPS and UM-3003 induced TNF, which polarizes Th1 cells (Fig. 5A–C). UM-3003, tested at up to 10 μM, significantly induced TNF in newborn MoDCs as compared to vehicle control (Fig. 5C). TLR8 is a vita-PAMP receptor that mediates IL-12 production and T_FH_ differentiation^22^. Consistent with our prior studies of other TLR7/8As^26,28^, UM-3003 induced IL-12p70 production in human adult (Fig. S5) and newborn MoDCs (Fig. 5A,B,D and Table S3B). Overall UM-3003 was an effective activator of human MoDCs in vitro.

To further characterize Th-polarizing cytokine induction, the ability of UM-3003 to induce Th1/Th2/Th17 response was tested after TCR stimulation in human neonatal PBMCs (Fig. 5E,F, Table S3C,D). The stimulator of interferon genes (STING) ligand 2′ 3′-cGAMP coordinates Th1-biased humoral and cell mediated immunity^54^. 2′ 3′-cGAMP along with the TLR7/8A CL075 served as positive controls for TCR stimulation^55^. UM-3003 induced robust IL-17 production after α-CD3 stimulation (Fig. 5E,F, Table S3D). In contrast, the control Th1 agonist 2′3′-cGAMP + CL075 induced a Th1 polarized response (Table S3C,D) with no increase in Th17. Induction of these Th1- and Th17-polarizing cytokines upon TCR stimulation highlights the potential of UM-3003 as a candidate adjuvant to enhance immunogenicity of an aP vaccine.

### Combined adjuvantation with TLR7/8A:alum drives neonatal Th1-polarized responsiveness to DTaP

**Figure 6:**
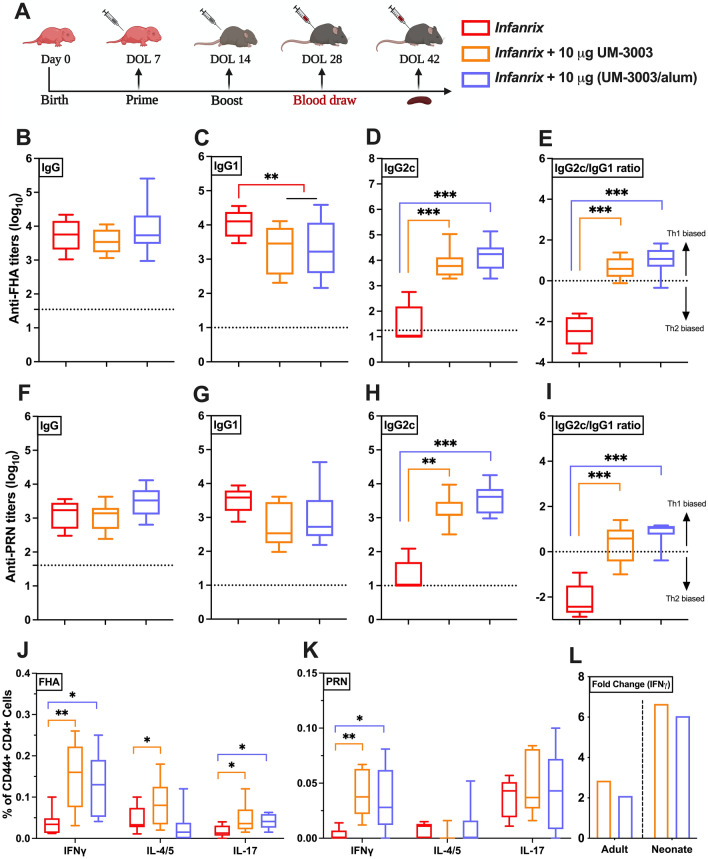
UM-3003 enhances DTaP specific IgG2c production and Th1/Th17 polarization in neonatal mice. (**A**) 7 days old C57BL/6 were vaccinated (prime/ boost) with DTaP (1/100th of the human dose) ± UM-3003 or UM-3003 at 10 µg per mouse in alum absorbed formulations. Serum was harvested 14 days following boost (DOL 28). Anti-FHA (**B**–**E**) and anti-PRN (**F**–**I**) serum total IgG (**B**, **F**) titers, IgG1 (**C**, **G**) and IgG2c (**D**, **H**) were measured by ELISA. PBS control groups were represented as dotted line. (**E**, **I**) IgG2c/IgG1 ratios for FHA and PRN respectively. (**J**, **K**) Splenic CD4^+^ T cell responses after FHA and PRN stimulation. Four weeks following booster, splenocytes from vaccinates were isolated and stimulated with 2 μg/ml of either FHA or PRN along with CD28 (1 μg/ml) and CD49d (1 μg/ml) for 12 h followed by 6 h of BFA stimulation. After stimulation, cells were harvested and stained (intracellular cytokine staining) and analyzed by flow cytometry. Plots were gated on CD44^+^ CD4^+^ lymphocytes and analyzed for all combinations of simultaneous IFNγ, IL-4/5 and IL-17A productivity. (**L**) Fold change of FHA specific IFNγ production in splenic CD4^+^ T cell relative to DTaP are shown. Statistical comparison was performed either using one-way ANOVA (C, E) or nonparametric Kruskal–Wallis test corrected for multiple comparisons; *p < 0.033, **p < 0.002, ***p < 0.001 (n = 9–13 per group), with comparison to DTaP alone. Study is inclusive of two independent repeats.

Having demonstrated the potential of alum:UM-3003 to enhance both immunogenicity of *Infanrix* and Ab subclasses induction in adult mice, we next assessed its activity in newborn mice. Of note, vaccines adjuvanted solely with alum typically drive Th2 responses at the expense of Th1 immunity and fail to induce Ab isotype switching toward IgG2c in early life^54^. 7 days old C57BL/6 were vaccinated with a prime-boost schedule (Fig. 6A) comprised of a two-injection series administered at Day of Life (DOL) 7 and 14 with DTaP (*Infanrix*) (1/100th of the human dose) ± UM-3001 (which did not induce adjuvanticity, Fig. S6) or UM-3003 at 10 µg per mouse. Serum was harvested 14 days following boost (at DOL 28) to measure Ab production (Fig. 6B-I). At two weeks post-secondary vaccination of DTaP (1/100th of human dose) with 10 µg of UM-3003, anti-FHA and anti-PRN IgG2c serum Ab titers were significantly elevated over DTaP alone (*p* < 0.001 and *p* < 0.002, respectively) or with additional alum (10 µg, *p* < 0.001) (Fig. 6D,H), with a Th1 biased IgG2c/IgG1 ratio (Fig. 6E,I). In addition, mice (both adult and infant) were bled four weeks after the booster immunization (Figs. 4A, 6A) to assess the kinetics of the antibody responses. Interestingly, alum adsorbed UM-3003 formulated with DTaP increased anti-FHA IgG (*p* < 0.002) and IgG2c (*p* < 0.001) responses in infant mice only on DOL 42 compared to the observed humoral responses of DOL 28 (Fig. S7E,F). Evaluation of infant FHA- and PRN-specific memory CD4^+^ T cell responses confirmed intracellular IFNγ production and thus Th1 polarization among DTaP:TLR7/8A immunized mice (Fig. 6J,K and S3). In addition, we observed formulated DTaP with UM-3003 induced FHA-specific polyfunctionality (Th2/Th17) responses in infant mice (Fig. 6J). As compared to DTaP alone, a highly consistent DTaP:TLR7/8A FHA-specific IFNγ secretion by neonatal splenocytes was also observed (Fig. S4G, *p* < 0.002). Remarkably, we demonstrate that, compared to DTaP alone, TLR7/8 adjuvantation increased FHA-specific IFNγ responses ~ 6–8-fold increase in infant mice, as compared to ~ 2–2.5-fold increase for their adult counterparts (Fig. 6L). These observations highlight the age-specific nature of the DTaP:TLR7/8A formulation and suggest that our in vitro Th1-polarizing leukocyte studies predict in vivo activity.

## Discussion

*B. pertussis* is an important cause of childhood morbidity and mortality, yet current vaccination strategies for newborns and infants are suboptimal. There is an unmet need to develop a more effective infant pertussis vaccine because of the resurgence of pertussis in many countries and the continued phenomena of waning immunity in older infants and adults^56^. Current alum-adjuvanted aP vaccines have shortcomings which may contribute to suboptimal infant responses^57^. New precision adjuvants, selected based on their activity towards a target age group, may enable development of next generation vaccines to optimize protection of vulnerable populations. Such an approach could in principle enhance early life immunogenicity by creating a vaccine formulation that induces both accelerated and robust immunity to *B. pertussis*. In the present study, we explored the adjuvant potential of imidazoquinoline small molecule TLR7/8 agonists by assessing their robust activity in inducing Th-polarizing cytokines from human newborn leukocytes in vitro, generating a phospholipidated derivative (UM-3003) and adsorbing it onto alum thereby generating a combined adjuvantation system (UM-3003/alum). In a neonatal mouse immunization model, addition of (UM-3003/alum) induced a balanced Th1/Th17-polarized cell mediated and IgG2c-skewed humoral response to a licensed acellular vaccine (*Infanrix*). TLR7/8A:alum represents a promising adjuvant combination that may enable an improved pertussis vaccine to optimally protect the very young.

The persistently high global burden of infections in the very young^58^ provides a compelling rationale for developing additional safe and effective early life vaccines. Small molecule TLR7/8As have demonstrated great potential as vaccine adjuvants^38,59^, since they directly activate antigen presenting cells and can enhance both humoral and cellular immune responses, especially Th1 responses^60^. Along with effective adjuvantation, vaccine delivery systems, improvement in antigen design and increased knowledge about human immune responses, are key technological advances fueling the current revolution in vaccine discovery and development^61^. Rational vaccine design approaches, employing immuno-engineering and novel delivery systems may enable controlled preparation of vaccine complexes with the desired immuno-stimulatory properties, particulate size, and antigen load, all of which also improve safety by potentially limiting systemic toxicities by their targeted nature^17,62^.

Overall, there are several synthetically designed TLR7/8 adjuvanted vaccine formulations at various stages of clinical development, which may offer significant advantages compared to current alum-based subunit vaccines consisting of complex and heterogeneous products. Benzonaphthyridine (BZN) TLR7 agonists have been chemically modified with phosphonates to enable adsorption onto alum hydroxide^59,63^. The adsorption of TLR7/8 adjuvants to alum shares some similar advantages to lipidation and encapsulation, such as (i) exploiting the inherent adjuvanticity of alum, (ii) facilitating simultaneous co-delivery of antigen and adjuvant, while also (iii) retaining the small molecule adjuvant at the site of vaccination, thereby limiting the systemic distribution of the adjuvant^59^. TLR7/8 agonists have demonstrated significantly enhanced adjuvanticity in the context of several licensed vaccine classes, including formulation with: recombinant group B meningococcus (MenB) proteins alone or with the full 4-component alum-adjuvanted MenB vaccine formulation (4CMenB, Trade name: *Bexsero*)^59^, the glycoconjugate CRM197-MenC vaccine (Trade name: *Menjugate*)^64^ and a tetanus, diphtheria, and acellular pertussis (whooping cough) (DTaP) vaccine^30^. Of note, TLR7/8 adjuvantation approaches have opened up an opportunity to shape responses to diseases that are often characterized as non-vaccine preventable^62^, including *Staphylococcus aureus* and HIV. A recent report by our group suggested that novel lipidated TLR7/8 agonists are effective adjuvants that can trigger influenza-virus specific Th1/Th17 polarization and humoral responses in mice^60^. In a phase I randomized, dose escalation clinical study of an AS37-adjuvanted meningococcal C conjugated vaccine (i.e., Alum + TLR7A) appears safe and well tolerated in adults^65^. This also extends to viral infections, where TLR7/8As can significantly improvement vaccine efficacy, leading to improved survival post challenge, such as for Severe Acute Respiratory Syndrome Coronavirus 2 (SARS-CoV-2) and influenza virus (H1N1) vaccines^66^. Continued evaluation and optimization of these TLR7 and TLR7/8 adjuvant approaches, including potential dose-sparing effects, decreased reactogenicity, increased safety, and efficacy profiles, are clearly merited^33^.

Our study synergistically combined three innovative approaches to provide unique insight into and overcome early life aP vaccine hyporesponsiveness: (1) use of age-specific human in vitro and murine in vivo models, (2) employing cutting-edge medicinal chemistry and (3) leveraging formulation science to optimize small molecule adjuvanticity and delivery. Next generation pertussis vaccines will likely require optimization of antigens and adjuvantation to enhance immunogenicity while maintaining relatively low reactogenicity.

The ability of UM-3003 to induce TNF and IL-12p70 production by neonatal MoDCs as well as Th17-polarization by neonatal T cells (albeit FHA-specific) is notable. Activated MoDCs initiate distinct signaling cascades and polarize CD4^+^ T helper cells. Acellular pertussis vaccine mostly induced Th2 polarization which may contribute to waning immunity^67,68^. As compared to other pharmacophores with TLR7/8 agonism, UM-3003 induces a more balanced Th-polarizing innate cytokine environment upon TCR stimulation. Our results raise the possibility that an appropriately formulated DTaP adjuvanted with UM-3003, which induces a Th1/17-response similar to that achieved by whole cell vaccination or natural pertussis infection, may induce immunity to prevent transmission of pertussis infection and long-term durable protective immunity. One unavoidable shortcoming worth highlighting of studies such as ours, that focus on TLR7/8 agonist adjuvant development, is the reduced applicability of pertussis challenge model in mice. IMQs trigger TLR7/8 pathway in a species-specific manner, with murine TLR8 being most distinct, and not activated by the majority of known human specific TLR8 agonists^33^. Interestingly, pig and non-human primate TLR8 innate immune responses are very similar to those observed in humans^33^. Therefore, it remains to be assessed whether Abs induced by UM-3003-adjuvanted DTaP can prevent infection with *B. pertussis *in vivo, a topic worthy of future study by using pigs and non-human primates. To provide a deeper insight into the role of TLR7/8 adjuvants in overcoming any potential waning responses post DTaP vaccination, future studies should also endeavor to evaluate sustained antibody production beyond four weeks after the last boost, especially in immunized infant animals.

In summary, we demonstrate that (a) UM-3003 robustly activates human neonatal MoDCs in vitro to produce IL-12p70 and (b) upon immunization of newborn mice in vivo, (UM-3003/alum) adjuvanted DTaP vaccination enhanced FHA- and PRN-specific Th1/Th17 cell mediated immunogenicity, including switched humoral responses toward the highly functional IgG2a/c subclass, which better mimics cellular immunity profiles triggered by natural *B. pertussis* infection and DTwP vaccination^69^. By combining age-specific early life human in vitro and mouse in vivo models, medicinal chemistry and formulation science, as well as benchmarking to licensed vaccines, our study provides a fresh paradigm for precision vaccine design to inform development of novel age-targeted neonatal and pediatric vaccines. This precision adjuvantation approach may be of fundamental importance in vaccine discovery and development and could represent a new paradigm for effective early life immunization against pertussis and other pathogens.

## Methods

### Ethics statements

All experiments were conducted in accordance with relevant institutional and national guidelines, regulations, approvals and in compliance with the ARRIVE (Animal Research: Reporting of In Vivo Experiments) guidelines. All experiments involving animals were approved by the Institutional Animal Care and Use Committees (IACUC) of Boston Children’s Hospital and Harvard Medical School (protocol numbers 19-02-3792R and 19-02-3897R) and the University of Montana (protocol number 037-16ASDBS-061416). C57BL/6 (either 6–8 weeks or pregnant) and BALB/c mice were obtained from Taconic Biosciences (Rensselaer, NY), Jackson Laboratories (Bar Harbor, ME) or Envigo (Indianapolis, IN) and housed in specific pathogen-free conditions in the animal research facilities at Boston Children’s Hospital or the University of Montana. For pregnant mice, cages checked daily to assess the presence of pups. Discovery of a new litter was recorded as DOL 0. Both male and female pups were used for neonatal experiments in a littermate-controlled specific manner. CO_2_ was used as the primary euthanasia method, with cervical dislocation as a secondary physical method to ensure death.

Non-identifiable human cord blood samples were collected with approval from the Ethics Committee of The Brigham & Women’s Hospital, Boston, MA (protocol number 2000-P-000117) and Beth Israel Deaconess Medical Center, Boston, MA (protocol number 2011P-000118). The requirement for informed consent was waived for the non-identifiable human cord blood samples. Blood samples from adult volunteers were collected after written informed consent with approval from the Ethics Committee of Boston Children’s Hospital, Boston, MA (protocol number X07-05-0223) or the University of Montana Institutional Review Board (protocol number 43-16).

### Human blood sample processing and in vitro stimulation

Peripheral blood was collected from healthy adult volunteers, while human newborn cord blood was collected immediately after Cesarean section delivery of the placenta. Births to known HIV-positive mothers were excluded. Human experimentation guidelines of the U.S. Department of Health and Human Services were observed at the Brigham & Women’s Hospital, Beth Israel Deaconess Medical Center, Boston, Boston Children’s Hospital and the University of Montana and following protocols were approved by the local institutional review boards.

Human blood was anti-coagulated with 20 units/ml pyrogen-free sodium heparin (American Pharmaceutical Partners, Inc.; Schaumberg, IL). All blood products were kept at room temperature (RT) and processed within 4 h from collection. Human whole blood assays were completed as previously described^25,28^. Briefly, neonatal cord blood or adult whole blood (WB) was mixed 1:1 with sterile pre-warmed (37 ℃) RPMI 1640 medium (Invitrogen; Carlsbad, CA) and 180–225 µl of the 1:1 suspension was added to each well of a 96 well U-bottom plate (Becton Dickinson; Franklin Lakes, NJ, USA) containing 20–25 µl of freshly prepared specific TLRAs at 10 × of the final concentration. Suspensions containing 200–250 µl/well were gently mixed by pipetting and incubated for 6 h at 37 °C in a humidified incubator at 5% CO_2_. After stimulation, plates were centrifuged at 500 × g and ~ 100–150 μl of supernatant was carefully removed by pipetting without disturbing the cell pellet. Supernatants derived from human leukocyte stimulations were assayed by ELISA for TNF and IFNγ (BD Biosciences; San Jose, CA, USA).

For adult PBMC and neonatal CBMC stimulation, primary human PBMCs and CBMCs were isolated from fresh blood via Ficoll gradient separation. PBMCs and CBMCs were resuspended and maintained in RPMI 1640 culture media (Invitrogen, Grand Island, NY) containing antibiotics (Pen/Step/Glut, Invitrogen) and 10% FBS (Sigma). Cells were plated at 0.1 × 10^6^ cells/well in 96-well tissue culture plates and stimulated for 24 h with aqueous formulations of indicated compounds. Culture supernatants were harvested and analyzed for IFNα and TNF induction using human TNF DuoSet (R) ELISA kit and human IFNα VeriKine ELISA kit (Pestka Biomedical Laboratories, Inc., Piscataway, NJ).

Additionally, for both newborn and adult in vitro assays, cytokine and chemokine expression profiles in cell culture supernatants and peripheral blood plasma were measured using customized Milliplex human cytokine/chemokine magnetic bead panels (Millipore; Billerica, MA, USA). Assays were analyzed on the Luminex® 100/200™ System employing xPOTENT® software (Luminex; Austin, TX) and Millipore Milliplex Analyst (version 3.5.5.0). The minimum threshold for each analyte was set at the minimum detectable concentration for a given assay, defined as three standard deviations above the mean background. CL075 (TLR8/7), R848 (TLR7/8) and 2′3′-cGAMP (STING) were purchased from InvivoGen (San Diego, CA) and used at the concentrations noted in the figure legends.

### Endotoxin evaluation and HEK293 assay for human TLR7 and TLR8 selectivity

All TLR7/8As used in both in vitro and in vivo studies were verified to be free of endotoxin (< 1 EU/ml) by the Endosafe nexgen-MCS *Limulus amoebocyte lysate* assay per the manufacturer’s instructions (Charles River Laboratories). Human embryonic kidney (HEK)293 cells expressing human TLR7 or TLR8 with an NF-κB-responsive secreted embryonic alkaline phosphatase (SEAP) reporter gene were obtained from Novus Biologicals (Littleton, CO) and InvivoGen (San Diego, CA), respectively. Cells were maintained in DMEM with 10% HI-FBS and selection antibiotics per the manufacturer’s instructions. Cells were plated at 5 × 10^5^ cells/96-well and stimulated with indicated agonist(s) for 24 h. Supernatants were harvested and analyzed for NF-κB/SEAP activation using the QuantiBlue kit (InvivoGen). Values are expressed as fold change in OD_650_ over vehicle-only treated samples.

### Human newborn Th1/Th17 polarization assay

Human newborn CBMCs, a mixed mononuclear cell culture in which the T cell compartment is largely composed of naïve T cells, were stimulated with the TLR7/8A and STING agonist (2′3′-cGAMP) in the presence of the polyclonal T cell activator α-CD3 (Plate-bound, 5 μg/ml) for 96 h. T cell polarization was evaluated by a high sensitivity T cell multiplex assay.

### Quantification of UM-3003 adsorption onto alum derived from DTaP vaccine

To quantify the extent of UM-3003 adsorption to aluminum hydroxide (Alhydrogel) we mixed 100 μl of UM-3003 with 100 μl of *Infanrix* (GSK) (combination vaccine for diphtheria, tetanus, and acellular pertussis (DTaP)) (a 1:10 UM-3003:alum mass ratio) plus 300 μl of 0.9% saline. After vortexing for 10 s the sample was placed in a 37 ℃ incubator. Every 15 min (minutes) the sample was vortexed for an additional 5 s and placed back into the incubator. Aliquots (0.75 ml) were taken at t =0,  0.25, 0.5, 1, 3 and 23 h and centrifuged at 3000 RPM (rcf = 664 g) to separate the alum from the supernatant. Supernatant was immediately removed and placed into an autosampler vial undiluted for analysis by reverse-phase high performance liquid chromatography (RP-HPLC) to determine adsorption as a function of time. RP-HPLC samples were run on a Waters 2695 HPLC equipped with a 2996 photodiode array detector at a wavelength of 254 nm. A gradient was performed using a two mobile phase system of 0.1% trifluoroacetic acid in water and 0.1% trifluoroacetic acid in acetonitrile, on an Agilent Zorbax Eclipse Plus C18, 4.6 × 150 mm, 5 micron column at 25 ℃. The response (peak area) of the samples were compared against a 50 μl UM-3003 plus 200 μl 0.9% saline control and a separate 100 μl alum plus 400 μl saline control.

Dynamic light scattering (DLS) analysis was performed using a Zetasizer Nano-ZS (Malvern Panalytical, USA) to measure the size and polydispersity index of UM-300X series. All samples were diluted 1 in 1000 with 2% glycerol prior to the analysis.

### In vitro release and desorption kinetics of UM-3003

In vitro release and desorption kinetics of UM-3003 from UM-3003 adsorbed alhydrogel formulation were performed using 100% adjuvant-free human plasma as the release medium to simulate in vivo conditions. For the in vitro release experiment, 50 µl of UM-3003 adsorbed alhydrogel formulation was mixed with 500 µl of adjuvant-free human plasma in a closed glass vial at 37 °C and gently stirred at 100 rpm. At different time points (0, 0.25, 0.5, 2, 4, 8, 12 and 24 h), 55 µl of the release medium was collected and replenished with a fresh 55 µl of adjuvant-free human plasma to simulate the in vivo sink conditions^70,71^. In vitro desorption experiments were carried out similarly to the in vitro release study protocol without stirring the release medium and without replenishing the release medium. Following in vitro release and desorption studies, the plasma samples were centrifuged at 8,500 rpm for 5 min to pellet the UM-3003 adsorbed alhydrogel formulation. The supernatant (50 µl), which contained desorbed UM-3003 was collected and retained at 2–8 °C until further analysis by RP-HPLC. The amount of UM-3003 present in the samples was quantified by RP-HPLC after extraction. To identify the mechanism of release of UM-3003 from the alhydrogel adsorbed formulation, the in vitro release profile was fitted with various kinetic models such as zero order, first order, Higuchi, Korsmeyer-Peppas and Hixson-Crowell using DD solver, using a Microsoft Excel plugin^72^ followed by Heat-Map transformation.

### In vivo rodent immunization, antigens and Ab quantification

For immunization experiments, both neonate and adult mice were immunized intramuscularly (i.m.) in the posterior thigh with 50 μl of total vaccine dose. For adult mouse studies, either BALB/c mice or C57BL/6 (6–8 weeks of age) were immunized with *Infanrix* (1/100th of the human dose) ± UM-3001 or UM-3003 at 0.1 μg, 1 μg or 10 μg per mouse in different formulations (aqueous choline salt or alum pre-adsorbed) or alum at 10 μg per mouse. Serum was harvested 14 days following prime (14dp1) or boost (14dp2) via retroorbital bleed and anti-FHA or anti-PRN serum Ab IgG, IgG1 and IgG2a/IgG2c titers were measured by ELISA, as described below. For neonatal mouse studies, 7 days old C57BL/6 mice were immunized with a prime-boost schedule (two injections, each one week apart, for newborn mice at DOL 7 and 14). Serum was harvested 14 days following boost (14dp2) (day of life 28) and anti-FHA or anti-PRN serum total IgG titers, IgG1 and IgG2c were measured by ELISA. To study the kinetics of anti-FHA specific humoral responses, mice (both adult and infant) were bled four weeks after the booster immunization. Mice were anaesthetized with 0.8 L per minute oxygen-delivered isoflurane during the immunization and retroorbital bleed procedures.

For anti-FHA or anti-PRN ELISAs, CoStar 96 well high-binding plates (Corning, Corning, NY) were coated with either 2 µg/ml FHA (BP-FHA-100, The Native Antigen Company/Cedarlane, Burlington, NC) or 1 µg/ml PRN (*B. pertussis* Pertactin, Strain: *B. pertussis* strain 165, The List Labs, Campbell, CA) in carbonate buffer pH 9.6, incubated overnight at 4 °C, washed 3 × with wash buffer (KPL 10X Phosphate Buffered Saline with 0.05% Tween 20 (Fisher Scientific)) and blocked with Superblock (ScyTek) for 1 h at RT. Then, sera from vaccinated mice were added with an initial dilution of 1:100 and 1:2 serial dilutions in EIA buffer (PBS + BSA 1% + Tween 20 0.1% + heat-inactivated FBS 10%) and incubated for 2 h at RT. Plates were then washed 3 × and incubated for 1 h at RT with HRP-conjugated anti-mouse IgG, IgG1, IgG2c or IgG2a (Southern Biotech). At the end of the incubation, plates were washed 5 × and developed with BD OptEIA TMB substrate reagent set (BD, San Jose, CA) for 10 min, then stopped with 1 M H_2_SO_4_. The optical density was measured at 450 nm with SpectraMax ID3 microplate reader with SoftMax Pro Version 5 (both from Molecular Devices) and either titers or concentrations were calculated using as cutoff three times the optical density of the background.

### Cell mediated immune responses in rodents

After two weeks post booster-immunization, mice spleens were collected in RPMI 1640 media containing 10% heat-inactivated FBS. For analysis of single-cell suspensions, spleens were mechanically and aseptically dissociated with the back of a syringe plunger and filtered through a 70-μm cell strainer and collected in RPMI 1640 media. After centrifugation (500 × g, 10 min, RT), cells were treated with 1 ml ACK lysis buffer (Gibco, Waltham, MA) for 2 min at RT to lyse red blood cells. Cells were washed immediately with RPMI 1640, passed through 70-μm cell strainer, and suspended in RPMI 1640 media (supplemented with 10% heat-inactivated FBS). Splenocytes were plated at a density of up to 2 × 10^6^ cells/well in a 96-well U-bottom plate and stimulated with 2 μg/ml of either FHA or PRN in T cell media. T cell media consists of RPMI 1640 (Gibco, Waltham, MA) supplemented with 10% heat-inactivated FBS (Cytiva HyClone, Fisher Scientific), 100 U/ml of Penicillin and 100 mg/ml of Streptomycin (Gibco, Waltham, MA), 55 mM of 2-mercaptoethanol (Gibco, Waltham, MA), 60 mM of  Non-essential Amino Acids (Gibco, Waltham, MA), 11 mM of HEPES (Gibco, Waltham, MA), and 800 mM of L-Glutamine (Gibco, Waltham, MA). In addition to antigen, the stimulation cocktail consisted of 1 μg/ml anti-mouse CD28/49d (BD Biosciences) as a co-stimulant.

For intracellular cytokine staining (ICS), 18 h stimulation was completed in a humidified incubator at 37 °C, 5% CO_2_ and 5 μg/ml of  Brefeldin A (BFA; BioLegend) was added during the last 6 h of stimulation, to block the cytokines production and facilitate optimal intracellular flow cytometry analysis. For quantification of antigen specific IFNγ and IL-17 responses by murine T cell multiplex assay, stimulation was done for 72–80 h of time period without any addition of BFA. Supernatants were collected and stored at − 80 °C for further analysis.

### Intracellular cytokine staining and flow cytometry

After 18 h of stimulation, cells were washed twice with PBS and blocked with Mouse Fc Block (BD Biosciences) according to the manufacturer’s instructions. After blocking, cells were washed once with PBS and stained with Aqua Live/Dead stain (Life Technologies, Carlsbad, CA) for 15 min at RT. Following two additional PBS washes, cells were resuspended in 100 ul of FACS buffer (PBS supplemented with 0.2% BSA (Sigma-Aldrich)) containing mouse specific cell surface markers for flow cytometry. Markers included anti-mouse CD44 PerCP-Cy5.5 and CD4 APC/Fire750^73^. Details of the clone and manufacturer of each marker used in a customized six colors flow-cytometry panel are documented in Supplementary Fig. S3C. Cells were incubated with the surface markers for 30 min at 4 ℃. Cells were washed with PBS and fixed/permeabilized by using the BD Cytofix/Cytoperm fixation/permeabilization solution kit according to the manufacturer’s instructions. Cells were washed in 1X perm/wash solution and subjected to intracellular staining (30 min at 4 °C) using a cocktail of the following Abs: anti-mouse IFNγ Alexa Fluor 488, IL-4/5 BV421 and IL-17A Alexa Fluor 647 in 1X perm/wash solution. Finally, cells were washed in PBS and fixed in PBS containing 1% paraformaldehyde (Electron Microscopy Sciences, Hatfield, PA) for 20 min at 4 ℃. After two final washes in PBS, the cells were resuspended in PBS and stored at 4 ℃ until acquisition. Samples were acquired on a BD LSR II (BD Biosciences; San Jose, CA) configured with blue (488 nm), yellow/green (568 nm), red (640 nm), violet (407 nm), and ultraviolet (355 nm) lasers using standardized good clinical laboratory practice procedures to minimize variability of data generated. Analysis was performed using FlowJo software, v.10.7.1 according to the gating strategy outlined in Supplementary Fig. S3A, B.

### Human Monocyte Derived Dendritic Cells (MoDC) assay

Monocytes were isolated from PBMC and CBMC fractions through positive selection by magnetic microbeads according to the manufacturer’s instructions (Miltenyi Biotec, Auburn, CA) using CD14 as a pan marker. Isolated monocytes were cultured in tissue culture dishes at 0.4 × 10^6^ cells/ml in RPMI 1640 media containing fresh 10% autologous plasma, supplemented with recombinant human IL-4 (50 ng/ml) and recombinant human GM-CSF (100 ng/ml) (R&D Systems, Minneapolis, MN) with one additional supplement of fresh media and cytokines at day 3 of culture as previously described^26,74^. After 6 days, immature MoDCs were harvested by gently pipetting the loosely adherent fraction, before being re-plated (10^5^ cells/ well) in 96-well flat-bottom plates in the presence or absence of TLRs, and/or 2% Glycerol, and/or sterile PBS. Plates were then incubated for 18–24 h at 37 ℃ in a humidified incubator at 5% CO_2_. After this stimulation supernatants were harvested and processed for further functional assays.

### Statistical analyses and graphics

Statistical significance and graphic output were generated using GraphPad Prism version 9 for macOS (GraphPad Software, La Jolla, CA, USA). Data were tested for normality by using the Shapiro–Wilk test. Group comparisons were performed by one- or two-way analyses of variance (ANOVAs) followed by post hoc Tukey’s test or Dunnett’s test for multiple comparisons. Measurements that failed normality tests were analyzed with Wilcoxon rank-sum tests or a Kruskal–Wallis rank-sum test followed by Dunn’s multiple comparisons within treatments groups. Results were considered significant at *p* values indicated in each figure legend. Sample size for mice experiments were chosen empirically based on the results of previous studies. Graphics in Figs. 3B, 4A, 6A, S1A, S2A and S6A were created with BioRender.com.

## Supplementary Information

Supplementary Information.

## Data Availability

The datasets generated and/or analyzed during the current study are available from the corresponding authors on reasonable request.

## References

[1] Cherry JD (2017). Treatment of Pertussis-2017. J. Pediatric Infect. Dis. Soc..

[2] Mattoo S, Cherry JD (2005). Molecular pathogenesis, epidemiology, and clinical manifestations of respiratory infections due to *Bordetella pertussis* and other *Bordetella subspecies*. Clin. Microbiol. Rev..

[3] Tan T, Trindade E, Skowronski D (2005). Epidemiology of pertussis. Pediatr. Infect. Dis. J..

[4] Burdin N, Handy LK, Plotkin SA (2017). What is wrong with pertussis vaccine immunity? the problem of waning effectiveness of pertussis vaccines. Cold Spring Harbor Perspect. Biol..

[5] Gu XX (2017). Waning immunity and microbial vaccines-workshop of the national institute of allergy and infectious diseases. Clin. Vaccine Immunol..

[6] Le T (2004). Immune responses and antibody decay after immunization of adolescents and adults with an acellular pertussis vaccine: The APERT Study. J. Infect. Dis..

[7] Heininger U, Cherry JD, Stehr K (2004). Serologic response and antibody-titer decay in adults with pertussis. Clin. Infect. Dis..

[8] Fouda GG, Martinez DR, Swamy GK, Permar SR (2018). The Impact of IgG transplacental transfer on early life immunity. Immunohorizons.

[9] Skoff TH, Baumbach J, Cieslak PR (2015). Tracking pertussis and evaluating control measures through enhanced pertussis surveillance, emerging infections program, USA. Emerg. Infect. Dis..

[10] Poolman JT (2014). Shortcomings of pertussis vaccines: Why we need a third generation vaccine. Expert Rev. Vaccines.

[11] Dowling DJ, Levy O (2015). Pediatric vaccine adjuvants: Components of the modern vaccinologist's toolbox. The Pediatric Infect. Dis. J..

[12] Dowling DJ, Levy O (2014). Ontogeny of early life immunity. Trends Immunol..

[13] Pollard AJ, Perrett KP, Beverley PC (2009). Maintaining protection against invasive bacteria with protein-polysaccharide conjugate vaccines. Nat. Rev. Immunol.

[14] Queenan AM (2019). Increasing FIM2/3 antigen-content improves efficacy of Bordetella pertussis vaccines in mice in vivo without altering vaccine-induced human reactogenicity biomarkers in vitro. Vaccine.

[15] Allen AC, Mills KH (2014). Improved pertussis vaccines based on adjuvants that induce cell-mediated immunity. Expert Rev. Vaccines.

[16] Dowling DJ, Levy O (2015). Pediatric vaccine adjuvants: Components of the modern vaccinologist's toolbox. Pediatr. Infect. Dis. J..

[17] Nanishi E, Dowling DJ, Levy O (2020). Toward precision adjuvants: Optimizing science and safety. Curr. Opin. Pediatr..

[18] Soni D (2020). Towards precision vaccines: Lessons from the second international precision vaccines conference. Front. Immunol..

[19] van Haren SD (2016). In vitro cytokine induction by TLR-activating vaccine adjuvants in human blood varies by age and adjuvant. Cytokine.

[20] Reed SG, Orr MT, Fox CB (2013). Key roles of adjuvants in modern vaccines. Nat. Med..

[21] Philbin VJ (2012). Imidazoquinoline Toll-like receptor 8 agonists activate human newborn monocytes and dendritic cells through adenosine-refractory and caspase-1-dependent pathways. J. Allergy Clin. Immunol..

[22] Ugolini M (2018). Recognition of microbial viability via TLR8 drives TFH cell differentiation and vaccine responses. Nat. Immunol..

[23] Browne EP (2012). Regulation of B-cell responses by Toll-like receptors. Immunology.

[24] Levy O (2006). The adenosine system selectively inhibits TLR-mediated TNF-alpha production in the human newborn. J. Immunol..

[25] Dowling DJ (2013). The ultra-potent and selective TLR8 agonist VTX-294 activates human newborn and adult leukocytes. PLoS ONE.

[26] Dowling DJ (2017). Toll-like receptor 8 agonist nanoparticles mimic immunomodulating effects of the live BCG vaccine and enhance neonatal innate and adaptive immune responses. J. Allergy Clin. Immunol..

[27] Holbrook BC (2017). An R848 adjuvanted influenza vaccine promotes early activation of B cells in the draining lymph nodes of non-human primate neonates. Immunology.

[28] Dowling DJ (2017). TLR7/8 adjuvant overcomes newborn hyporesponsiveness to pneumococcal conjugate vaccine at birth. Science.

[29] Pettengill MA (2016). Distinct TLR-mediated cytokine production and immunoglobulin secretion in human newborn naive B cells. Innate Immun..

[30] Misiak A (2017). Addition of a TLR7 agonist to an acellular pertussis vaccine enhances Th1 and Th17 responses and protective immunity in a mouse model. Vaccine.

[31] Soni D, Bobbala S, Li S, Scott EA, Dowling DJ (2020). The sixth revolution in pediatric vaccinology: Immunoengineering and delivery systems. Pediatr. Res..

[32] Brito LA, Malyala P, O'Hagan DT (2013). Vaccine adjuvant formulations: A pharmaceutical perspective. Semin. Immunol..

[33] Dowling DJ (2018). Recent advances in the discovery and delivery of TLR7/8 agonists as vaccine adjuvants. Immunohorizons.

[34] Vasilakos JP, Tomai MA (2013). The use of Toll-like receptor 7/8 agonists as vaccine adjuvants. Expert Rev. Vaccines.

[35] Sauder DN, Smith MH, Senta-McMillian T, Soria I, Meng TC (2003). Randomized, single-blind, placebo-controlled study of topical application of the immune response modulator resiquimod in healthy adults. Antimicrob. Agents Chemother..

[36] Szeimies RM (2008). A phase II dose-ranging study of topical resiquimod to treat actinic keratosis. Br. J. Dermatol..

[37] Dowling DJ (2017). TLR7/8 adjuvant overcomes newborn hyporesponsiveness to pneumococcal conjugate vaccine at birth. JCI Insight.

[38] Kasturi SP (2020). 3M–052, a synthetic TLR-7/8 agonist, induces durable HIV-1 envelope-specific plasma cells and humoral immunity in nonhuman primates. Sci. Immunol..

[39] Smith AJ (2016). Evaluation of novel synthetic TLR7/8 agonists as vaccine adjuvants. Vaccine.

[40] Gerster JF (2005). Synthesis and structure-activity-relationships of 1H-imidazo[4,5-c]quinolines that induce interferon production. J. Med. Chem..

[41] Evans JT (2019). Synthetic toll-like receptors 7 and 8 agonists: Structure-activity relationship in the oxoadenine series. ACS Omega.

[42] Bazin HG (2020). Optimization of 8-oxoadenines with toll-like-receptor 7 and 8 activity. Bioorg. Med. Chem. Lett..

[43] Levy, O., Dowling. D., Bazin, H.G., Burkhart, D., Evans, J., Smith, A.J. Methods and compositions relating to adjuvants. U.S. patent 20200108139 (2020).

[44] Pettengill MA, van Haren SD, Levy O (2014). Soluble mediators regulating immunity in early life. Front. Immunol..

[45] Mastelic B (2013). Predictive markers of safety and immunogenicity of adjuvanted vaccines. Biol. J. Int. Assoc. Biol. Standard..

[46] Horscroft NJ, Pryde DC, Bright H (2012). Antiviral applications of Toll-like receptor agonists. J. Antimicrobial Chemother..

[47] Strominger NL, Brady R, Gullikson G, Carpenter DO (2001). Imiquimod-elicited emesis is mediated by the area postrema, but not by direct neuronal activation. Brain Res. Bull..

[48] Lee J (2003). Molecular basis for the immunostimulatory activity of guanine nucleoside analogs: Activation of Toll-like receptor 7. Proc. Natl. Acad. Sci. USA.

[49] Chan M (2009). Synthesis and immunological characterization of toll-like receptor 7 agonistic conjugates. Bioconjugate Chem..

[50] Bazin HG, Bess LS, Livesay MT, Mwakwari SC, Johnson DA (2016). Phospholipidation of TLR7/8-active imidazoquinolines using a tandem phosphoramidite method. Tetrahedr. Lett..

[51] Bazin, H. G., Johnson, D.A. Pegylated Imidazoquinolines as TLR7 and TLR8 Agonists. U.S. patent 10,882,876 (2017).

[52] HogenEsch H, O'Hagan DT, Fox CB (2018). Optimizing the utilization of aluminum adjuvants in vaccines: You might just get what you want. NPJ Vaccines.

[53] Bruschi ML (2015). Strategies to Modify the Drug Release from Pharmaceutical Systems.

[54] Borriello F (2017). Identification and characterization of stimulator of interferon genes as a robust adjuvant target for early life immunization. Front. Immunol..

[55] Collier MA (2018). Acetalated dextran microparticles for codelivery of STING and TLR7/8 agonists. Mol. Pharm..

[56] Libster R, Edwards KM (2012). Re-emergence of pertussis: What are the solutions?. Expert Rev. Vaccines.

[57] Martinon-Torres F, Heininger U, Thomson A, von Wirsing KCH (2018). Controlling pertussis: How can we do it? A focus on immunization. Expert Rev. Vaccines.

[58] Liu L (2012). Global, regional, and national causes of child mortality: An updated systematic analysis for 2010 with time trends since 2000. Lancet.

[59] Wu TY (2014). Rational design of small molecules as vaccine adjuvants. Sci. Transl. Med..

[60] Miller SM (2020). Novel lipidated imidazoquinoline TLR7/8 adjuvants elicit influenza-specific Th1 immune responses and protect against heterologous H3N2 influenza challenge in mice. Front. Immunol..

[61] Koff WC (2013). Accelerating next-generation vaccine development for global disease prevention. Science (New York N.Y.).

[62] Delany I, Rappuoli R, De Gregorio E (2014). Vaccines for the 21st century. EMBO Mol. Med..

[63] Cortez A (2016). Incorporation of phosphonate into benzonaphthyridine toll-like receptor 7 agonists for adsorption to aluminum hydroxide. J. Med. Chem..

[64] Buonsanti C (2016). Novel adjuvant Alum-TLR7 significantly potentiates immune response to glycoconjugate vaccines. Sci. Rep..

[65] Gonzalez-Lopez A (2019). Adjuvant effect of TLR7 agonist adsorbed on aluminum hydroxide (AS37): A phase I randomized, dose escalation study of an AS37-adjuvanted meningococcal C conjugated vaccine. Clin. Immunol..

[66] Jangra S (2021). Sterilizing immunity against SARS-CoV-2 infection in mice by a single-shot and lipid amphiphile imidazoquinoline TLR7/8 agonist-adjuvanted recombinant spike protein vaccine. Angew Chem. Int. Ed. Engl..

[67] Mills KH, Ross PJ, Allen AC, Wilk MM (2014). Do we need a new vaccine to control the re-emergence of pertussis?. Trends Microbiol..

[68] Kapil P, Merkel TJ (2019). Pertussis vaccines and protective immunity. Curr. Opin. Immunol..

[69] Warfel JM, Zimmerman LI, Merkel TJ (2014). Acellular pertussis vaccines protect against disease but fail to prevent infection and transmission in a nonhuman primate model. Proc. Natl. Acad. Sci. USA.

[70] Kuijpers AJ (2000). In vivo and in vitro release of lysozyme from cross-linked gelatin hydrogels: A model system for the delivery of antibacterial proteins from prosthetic heart valves. J. Control Release.

[71] Northrup L (2017). Co-delivery of autoantigen and dexamethasone in incomplete Freund's adjuvant ameliorates experimental autoimmune encephalomyelitis. J. Control Release.

[72] Zhang Y (2010). DDSolver: An add-in program for modeling and comparison of drug dissolution profiles. AAPS J..

[73] Raeven RHM (2018). Molecular and cellular signatures underlying superior immunity against Bordetella pertussis upon pulmonary vaccination. Mucosal. Immunol..

[74] Ganapathi L (2015). The imidazoquinoline toll-like receptor-7/8 agonist hybrid-2 potently induces cytokine production by human newborn and adult leukocytes. PLoS ONE.

[75] Smith AJ (2016). Evaluation of novel synthetic TLR7/8 agonists as vaccine adjuvants. Vaccine.

[76] Bazin HG (2015). Structural requirements for TLR7-selective signaling by 9-(4-piperidinylalkyl)-8-oxoadenine derivatives. Bioorganic Med. Chem. Lett..

