# Supplementary material for: Development of a TLR7/8 agonist adjuvant formulation to overcome early life hyporesponsiveness to DTaP vaccination
Source: Sci Rep. 2022 Oct 18;12:16860. doi: 10.1038/s41598-022-20346-w (PMC9579132; doi:10.1038/s41598-022-20346-w)
Supplement: Supplementary file 1 — Supplementary Information. [file 41598_2022_20346_MOESM1_ESM.pdf]

SUPPORTING INFORMATION

Development of a TLR7/8 agonist adjuvant formulation to overcome early life hyporesponsiveness to DTaP vaccination

David J. Dowling[1,2,*], Soumik Barman[1,2,*], Alyson J. Smith[3*ψ], Francesco Borriello[1,2,4,5,ω], Danielle Chaney[1,2], Spencer E. Brightman[1], Gandolina Melhem[1], Byron Brook[1,2], Manisha Menon[1], Dheeraj Soni[1,2], Simone Schüller[1,2], Karthik Siram[6], Etsuro Nanishi[1,2], Hélène G. Bazin[3,6], David J. Burkhart[3,6], Ofer Levy[1,2,7**#], Jay T. Evans[3,6,**#]

[1]*Precision Vaccines Program*, Division of Infectious Diseases, Boston Children's Hospital, Boston, MA, USA, [2]Harvard Medical School, Boston, MA, USA, [3]Center for Translational Medicine, University of Montana, Missoula, MT, USA, [4]Department of Translational Medical Sciences, Center for Basic and Clinical Immunology Research (CISI), University of Naples Federico II, [5]WAO Center of Excellence, Naples, Italy, [6]Department of Biomedical and Pharmaceutical Sciences, University of Montana, Missoula, MT, USA, [7]Broad Institute of MIT & Harvard, Cambridge, MA, USA

[ψ]Current Address: Seagen (Bothell, WA, USA)

[ω]Current Address: Generate Biomedicines (Cambridge, MA, USA)

*D.J.D., S.B., and A.J.S. contributed equally to this work.

**O.L. and J.T.E. contributed equally to this work.

[#]Correspondence: O.L. [ofer.levy@childrens.harvard.edu] and J.T.E [jay.evans@mso.umt.edu]

INDEX

Supplementary Figures		Page
Figure S1	Imidazoquinoline UM-3001 induces IFNγ production in neonatal CBMCs.	S-3
Figure S2	UM-3003 enhances DTaP induced Ab production in adult mice in a titratable manner.	S-4
Figure S3	Gating strategy to identify FHA- and PRN-specific $CD4^+$ T cells.	S-5
Figure S4	UM-3003 enhances DTaP specific IFNγ recall responses in adult and infant mice.	S-6
Figure S5	UM-3001 and UM-3003 demonstrate concentration-dependent cytokine potency in newborn and adult human MoDCs.	S-7
Figure S6	Formulation of UM-3001 with the DTaP did not enhance Ab production in neonatal mice.	S-8
Figure S7	UM-3003 enhances FHA specific humoral responses in infant mice during post-vaccination.	S-9

Supplementary Tables		Page
Table S1	Cytokine profiling in human newborn cord blood after imidazoquinoline UM-3001 stimulation.	S-10
Table S2	Core compound UM-3001 does not adsorb to the alum/antigen.	S-11
Table S3	Cytokine profiling in human newborn MoDCs and CBMCs after lipidated imidazoquinoline UM-3003 stimulation.	S-12

Supplementary Figures

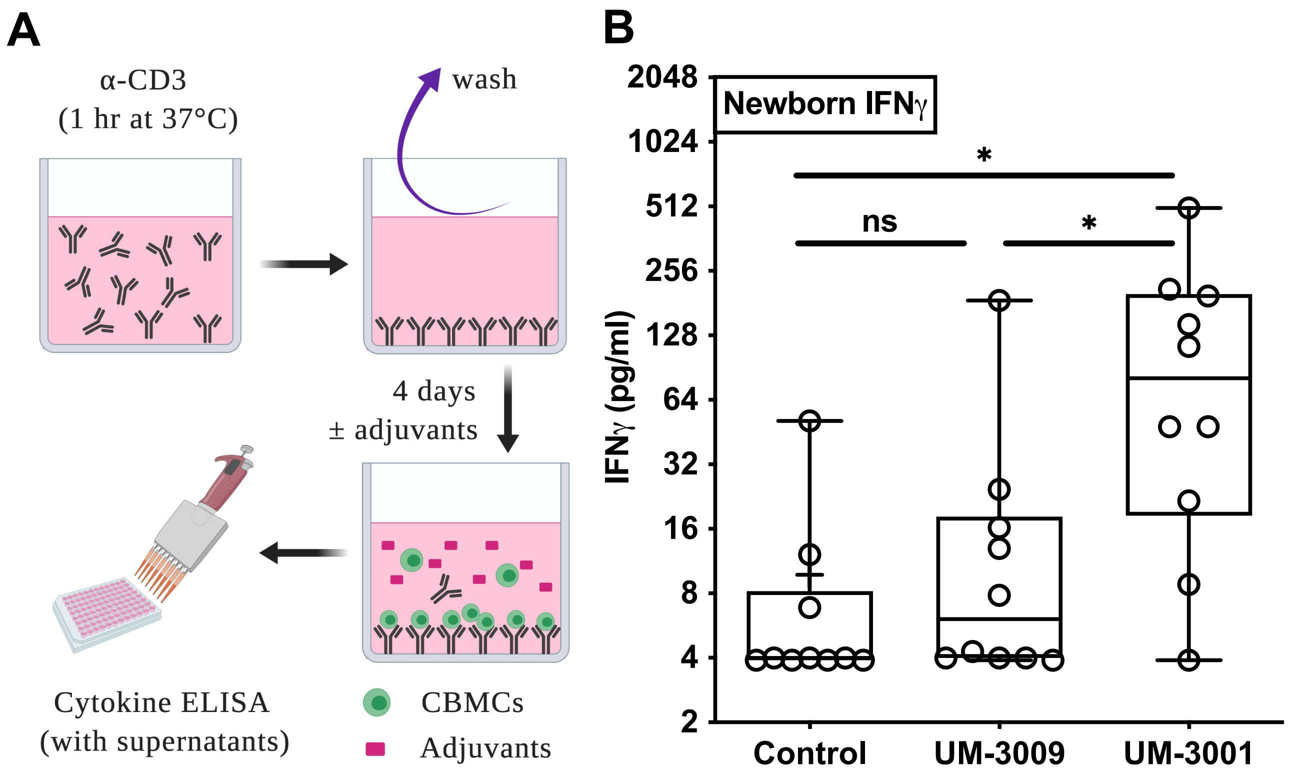

Figure S1. Imidazoquinoline UM-3001 induces IFNγ production in neonatal CBMCs. (A) Graphical representation of newborn CBMCs were stimulated with either UM-3009 or UM-3001, in the presence of the polyclonal T cell activator αCD3 (5 µg/ml) for 96 h. IFNγ levels were measured in cell-free supernatants by ELISA. (B) Results are depicted as the median, the 25th and 75th percentiles (boxes) and the 5th and 95th percentiles (whiskers) of 10 independent experiments. Statistical analysis was performed using nonparametric Kruskal-Wallis test followed by Dunn's multiple comparisons test; *$p < 0.033$.

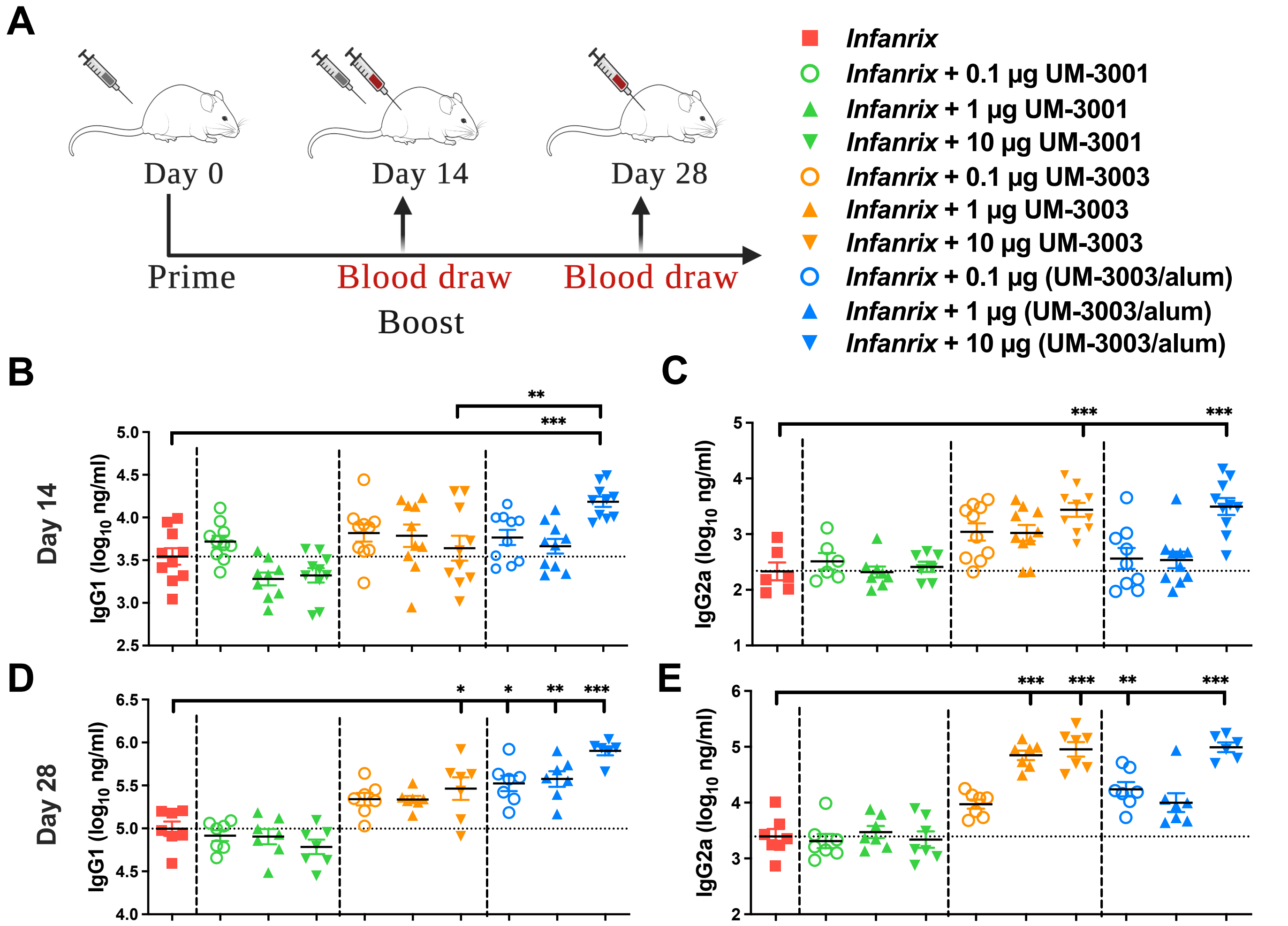

Figure S2. UM-3003 enhances DTaP induced Ab production in adult mice in a titratable manner. Adult BALB/c mice were immunized twice, 14 days apart (A) with DTaP (1/100th of the human dose) ± UM-3001 or UM-3003 at 0.1 μg, 1 μg or 10 μg per mouse in different formulations (aqueous or pre-adsorbed to alum). Serum was harvested 14 days following prime (B-C) or boost (D-E) and anti-FHA serum Ab IgG1 and IgG2a titers were measured by ELISA. Saline control groups were below the lowest limit of detection. Dotted line represented DTaP immunized group. Statistical comparison was employed by one-way ANOVA followed by either Dunnett's (when compared to *Infanrix*) or Tukey's post hoc test for multiple comparisons; *$p < 0.033$, **$p < 0.002$, ***$p < 0.001$ ($n = 6$ - 10 per group). Study was representative of two separate repeats.

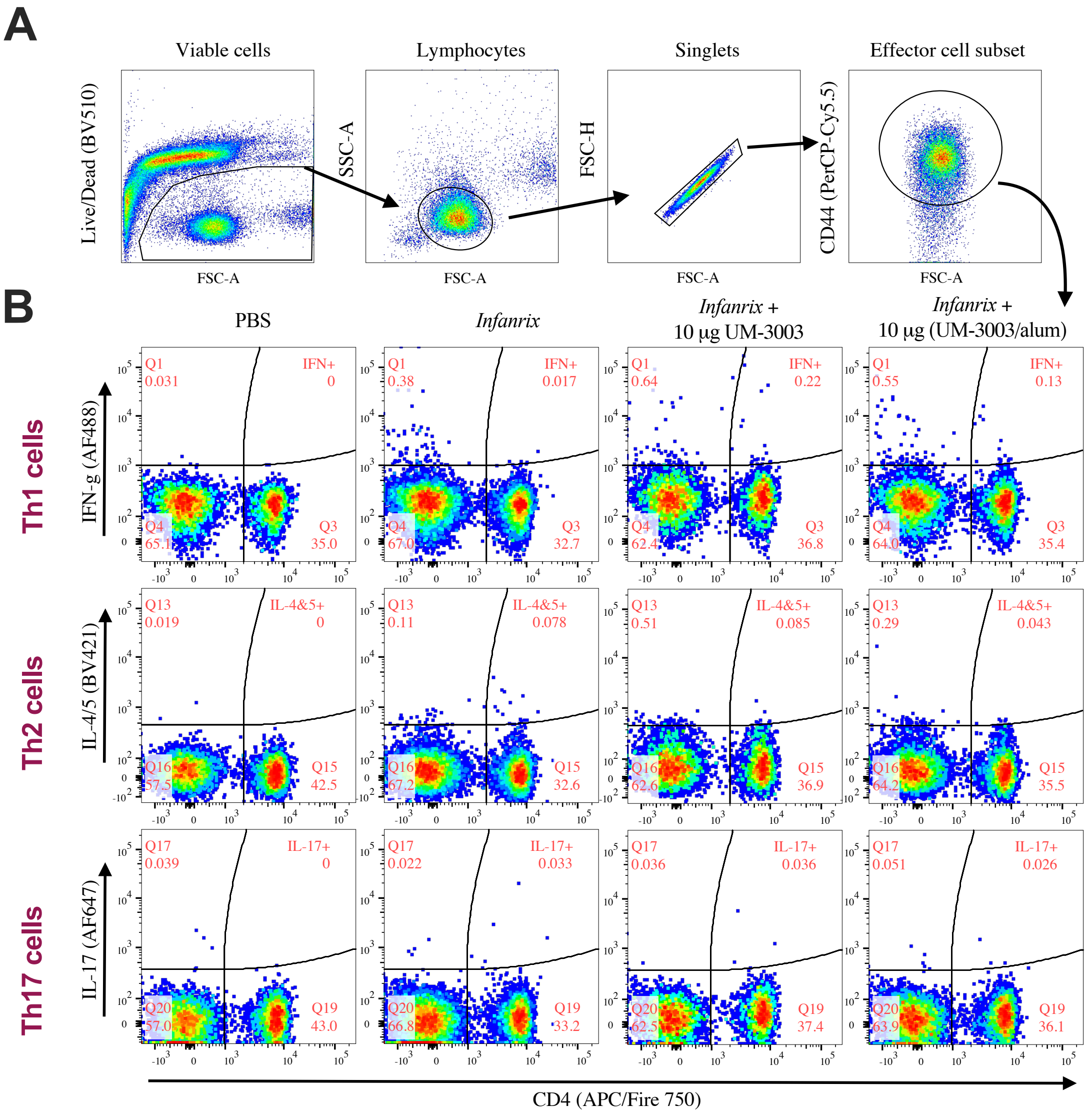

C

Assay	Target	Clone	Fluorochrome	Vendor	Catalogue No.	Titer
	Viability	---	Brilliant Violet 510	Invitrogen	L34966	1:500
	CD44	IM7	PerCP-Cy5.5	BioLegend	103032	1:160
T-Cell	CD4	RM4-5	APC/Fire 750	BioLegend	100568	1:160
Cytokines	IFN-γ	XMG1.2	Alexa Fluor 488	BioLegend	505813	1:160
	IL-4	11B11	Brilliant Violet 421	BioLegend	504119	1:40
	IL-5	TRFK5	Brilliant Violet 421	BioLegend	504311	1:160
	IL-17A	TC11-18H10.1	Alexa Fluor 647	BioLegend	506912	1:160

Figure S3. Gating strategy to identify FHA- and PRN-specific CD4$^+$ T cells. Splenocytes were isolated from immunized mice following four weeks booster. Antigen-specific T cell responses following FHA/PRN stimulation were defined as CD44^{hi}/CD4^{hi}/cytokine$^+$ using FlowJo software, v.10.7.1. (A) Shown is an example of the hierarchical gating strategy leading to the identification of live, singlet, CD44$^+$, CD4$^+$ and cytokine$^+$ T cells (B) with an example for IFNγ, IL-4/5 and IL-17 responses post vaccination with DTaP ± UM-3003. (C) Reagents for Flow Cytometric Analysis. Murine CD4$^+$ T cell compartment was dissected by a customized six colors flow-cytometry panel.

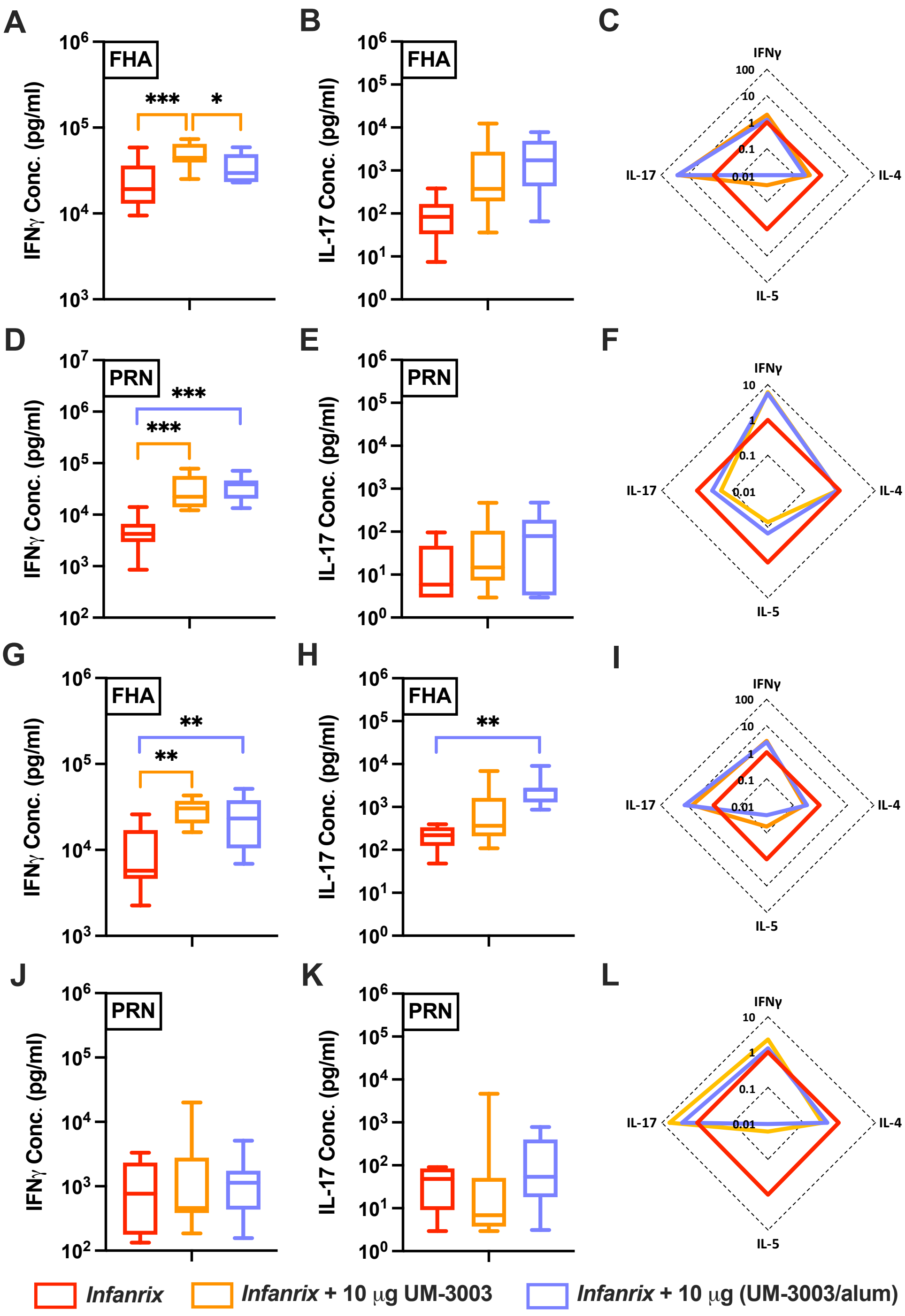

Figure S4. UM-3003 enhances DTaP specific IFNγ recall responses in adult and infant mice. Splenocytes were isolated from immunized adult (A-F) or infant (G-L) C57BL/6 mice following four weeks booster and stimulated with 2 µg/ml of either FHA or PRN along with CD28 (1 µg/ml) and CD49d (1 µg/ml) for 72 - 80 h. After stimulation, supernatants were collected for multiplex assay. FHA specific IFNγ (A, G), IL-17 (B, H) responses (pg/ml) and PRN specific IFNγ (D, J) along with IL-17 (E, K) responses (pg/ml) were represented (n = 8-15 for adult and 5-11 for infant mice). (C, F, I, L) Data are shown as fold change for adjuvanted group over DTaP immunized group (red line). For comparisons between overall groups, either one-way ANOVA or nonparametric Kruskal-Wallis test was applied, and statistical significance denoted as *$p < 0.033$, **$p < 0.002$ and ***$p < 0.001$.

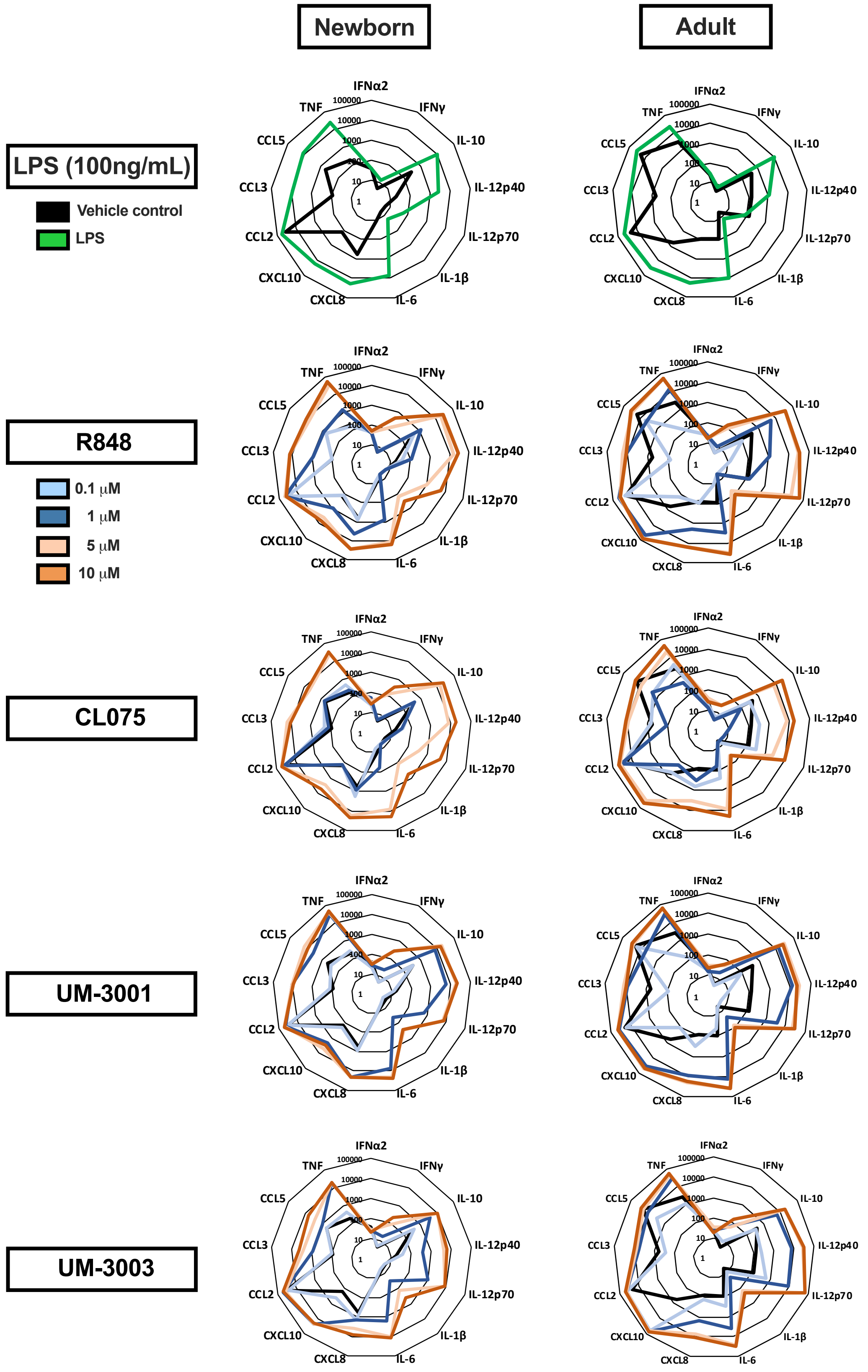

Figure S5. UM-3001 and UM-3003 demonstrate concentration-dependent cytokine potency in newborn and adult human MoDCs. Newborn (n = 4 - 5) and adult (n = 6) MoDCs were stimulated for 24h and supernatants were collected for multiplex assay. Cytokine responses of 13 analytes were represented as a radar plot. TLR4 agonist LPS (100 ng/ml), TLR7/8 agonists R848 and CL075 were employed as benchmarks. 2% glycerin in PBS served as a vehicle control.

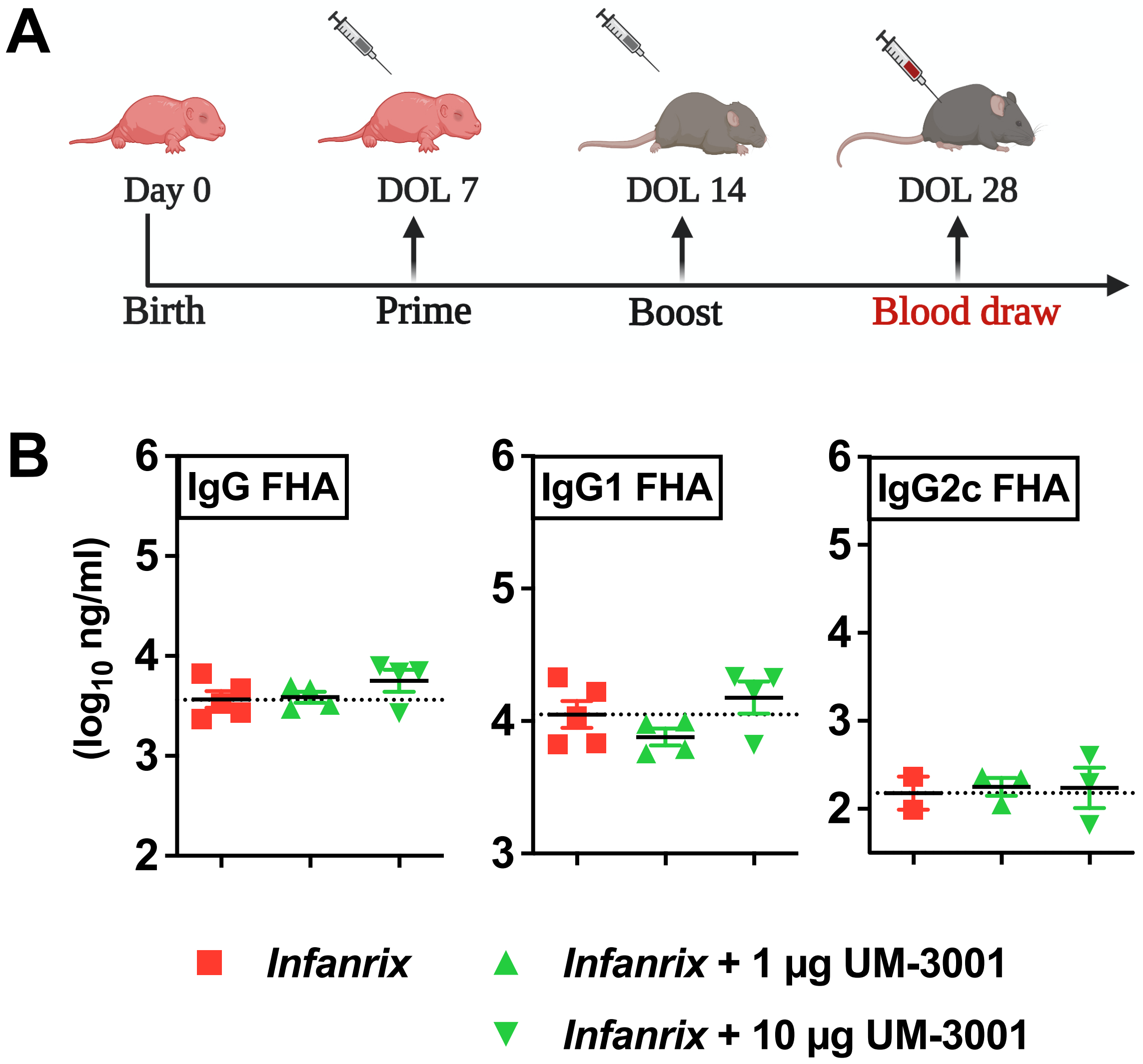

Figure S6. Formulation of UM-3001 with the DTaP did not enhance Ab production in neonatal mice. (A) 7 days old C57BL/6, 4 - 5 per group, were vaccinated (prime/ boost) with DTaP (1/100th of the human dose), ± UM-3001 at 1 µg or 10 µg per mouse. Serum was harvested 14 days following boost (14dp2) (DOL 28) and (B) anti-FHA serum total IgG titers, IgG1 and IgG2c were measured by ELISA. Dotted line represented DTaP immunized group. Statistical comparison employed nonparametric Kruskal-Wallis with comparison to DTaP alone.

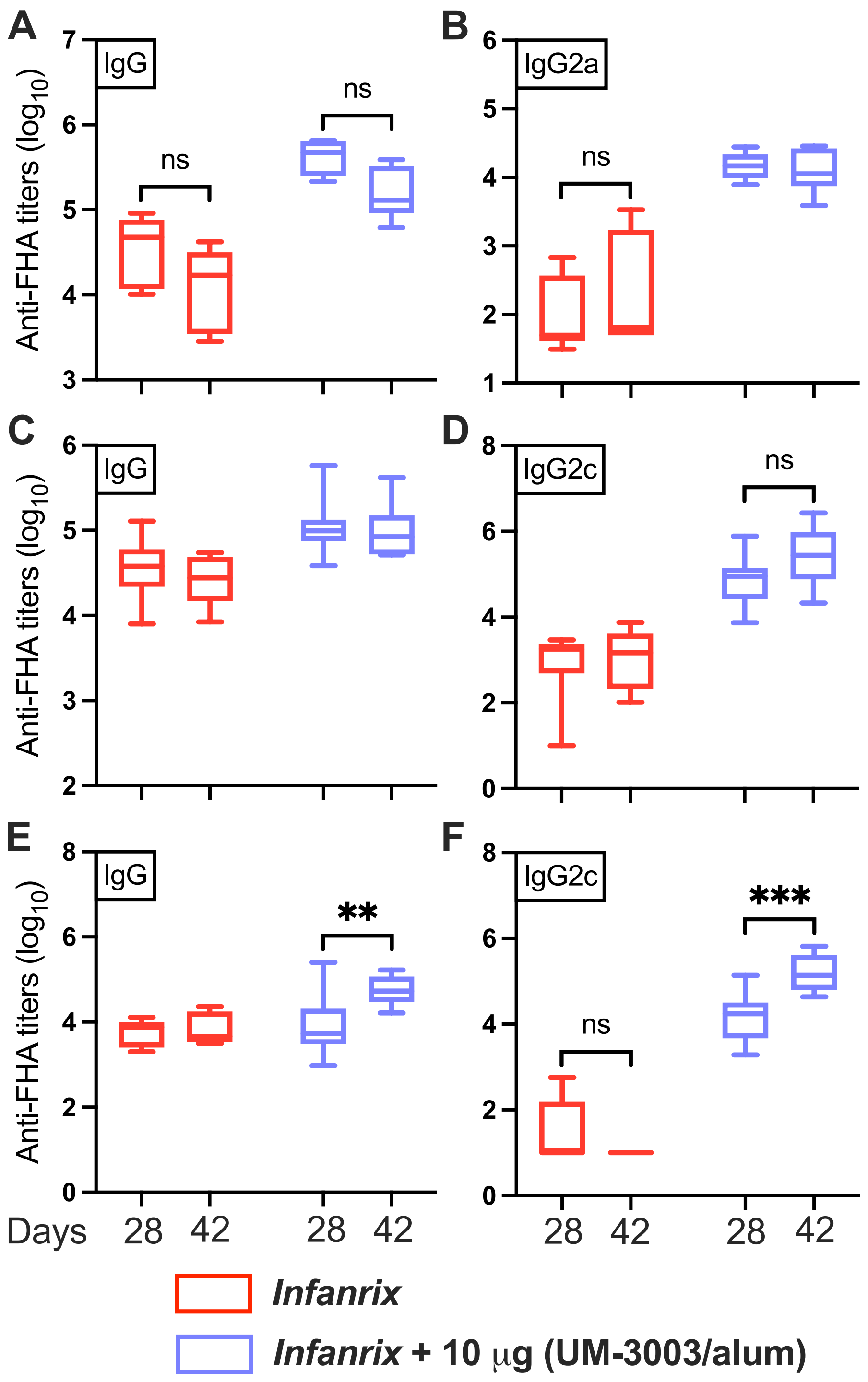

Figure S7. UM-3003 enhances FHA specific humoral responses in infant mice during post-vaccination. Adult BALB/c (A, B), adult C57BL/6 (C, D) and 7 days old C57BL/6 mice (E, F) were immunized twice, either 14 days apart (A-D) or 7 days apart (E, F) with DTaP (1/100th of the human dose) ± UM-3003 or alum at 10 µg per mouse in alum absorbed formulations. Serum was harvested 14 and 28 days following boost. Anti-FHA serum Ab IgG and IgG2c titers were measured by ELISA. Study (C-F) was inclusive of two independent repeats (n = 5 - 11 per group) where (A, B) was from one (n = 5 per group). Statistical comparison was determined by unpaired two-sided Wilcoxon rank-sum test where $^{**}p < 0.002$, $^{***}p < 0.001$ and ns denoted non-significant.

Supplementary Table 1: Cytokine profiling in human newborn cord blood after imidazoquinoline UM-3001 stimulation.

Data are shown as mean value of cytokine responses (pg/ml) for stimulated newborn cord or adult whole blood by 1 µM and 10 µM of UM-3001, (n = 6 adults, n = 8 newborns). Newborn cord and adult blood were cultured separately *in vitro* for 6 h with UM-3001. Supernatants were collected for multiplex assay. All significance determined by unpaired two-sided Wilcoxon rank-sum test where *P < 0.033 and **P < 0.002.

Agonist	Cytokine	Newborn (1 µM Stim)	Adult (1 µM Stim)	Fold change over adult	P-value	Newborn (10 µM Stim)	Adult (10 µM Stim)	Fold change over adult	P-value
	IFNα2	194.01	70.85	2.74	0.9677	209.86	270.5	0.78	0.2824
	IFNγ	150.96	62.78	2.40	0.4908	1685.55	419.61	4.02	0.01 (*)
	IL-10	4379.89	293.62	14.92	0.0013 (**)	3845.275	389.33	9.88	0.0127 (*)
	IL-12p40	1805	146.88	12.29	0.1812	2567.895	623.41	4.12	0.0813
	IL-12p70	6.46	10.19	0.63	0.1908	25.70625	67.38	0.38	0.0813
**UM-3001**	IL-1β	17068.02	861.53	19.81	0.0127 (*)	51416.155	7105.1	7.24	0.0107 (*)
	IL-6	21527.69	3964.53	5.43	0.0127 (*)	31750.9625	17303.67	1.83	0.0383 (*)
	CXCL8	6198.77	527.87	11.74	0.0290 (*)	7848.555	1176.72	6.67	0.0180 (*)
	CXCL10	32850.67	9806.11	3.35	0.2071	28708.1625	7350.39	3.91	0.1665
	CCL2	39835.74	4519	8.82	0.0113 (*)	42089.6125	3840	10.96	0.0043 (*)
	CCL3	3750.39	3583.61	1.05	0.4182	3750.395	5048.56	0.74	0.2774
	CCL5	35472.25	6433.33	5.51	0.0813	34435	7075.33	4.87	0.1029
	TNF	30669.25	3766.6	8.14	0.0196 (*)	60554.625	20139.5	3.01	0.0070 (*)
	GM-CSF	3.66	1.95	1.88	0.0093 (*)	5.765	2.228	2.59	0.0093 (*)

Supplementary Table 2: Core compound UM-3001 does not adsorb to the alum/antigen.

Direct adsorption of UM-3001 (MW of 320.82) to aluminum hydroxide derived from DTaP (*Infanrix*) vaccine, which has pertussis antigen adsorbed to its surface, was evaluated at 1, 2 and 24 h. The core UM-3001 compound demonstrated only minimal adsorption to the antigen (~ 4 - 7 % within 1-2 h), with peak area intensity levels similar to the unmixed controls, mAU: milli-absorbance units.

Sample	Time Point	Peak Area	% Adsorbed
**UM-3001:** ***Infanrix***	1h	114.30	-5.35%
	2h	101.20	6.73%
	24h	112.20	-30.92%
**UM-3001: Alum:** ***Infanrix***	1h	104.20	3.96%
	2h	107.80	0.65%
	24h	88.30	-3.03%
**Alum control**	0 (<5min)	n.a.	0.0%
	23h	n.a.	0.0%
**UM-3001 control**	0 (<5min)	108.50	
	24h	85.70	

Supplementary Table 3: Cytokine profiling in human newborn MoDCs and CBMCs after lipidated imidazoquinoline UM-3003 stimulation.

(A, B) Newborn MoDCs were stimulated for 24 h and supernatants were collected for multiplex assay. Data are shown as mean value of cytokine responses (pg/ml) of 13 analytes (n = 5).

(C, D) CBMCs were stimulated in the presence of the polyclonal T cell activator α-CD3 (5 μg/ml) in combination or alone for 96 h. TLR7/8 agonist CL075 (10 μM) and STING agonist 2'3'-cGAMP (10 μM) in combination were used as a benchmark control. Data are shown as mean value of cytokine responses (pg/ml) of 5 analytes (n = 6).

Statistical analysis was performed either using one-way ANOVA or nonparametric Kruskal-Wallis test corrected for multiple comparisons; $^{*}p < 0.033$ and $^{**}P < 0.002$.

Supplementary Table 3A:

Agonist	Cytokine	Vehicle control	LPS (100 ng/ml)	Newborn MoDCs (10 μM Stim)
UM-3003	IFNα2	37.86	36.452	20.22
	IFNγ	4.79	12.97	197.77
	IL-10	301.38	10123.04	10769.33
	IL-12p40	17.4	2598.98	6447.24
	IL-12p70	5.84	54.8	8092.06
	IL-1β	5.6	17.36	396.45
	IL-6	11.86	7053.29	11085.83
	CXCL8	612.42	19928.54	7625.18
	CXCL10	141.07	17119.7	21104.1
	CCL2	33225	52034.4	47392
	CCL3	89.07	8411.45	4008.42
	CCL5	521.65	13654.7	6449.32
	TNF	186.4	23888.56	18441.62

Supplementary Table 3B:

Agonist	Cytokine	Fold-change of LPS over vehicle control	P-value (LPS vs. vehicle control)	Fold-change of UM-3003 over vehicle control	P-value (UM-3003 vs. vehicle control)	P-value (LPS vs. UM-3003)
UM-3003	TNF	128.16	0.009 (*)	98.93	0.02 (*)	0.82
	IL-12p70	9.39	0.13	1385.63	0.01 (*)	0.33
	IL-1β	3.1	0.07	70.79	0.12	0.75
	IFNγ	2.7	0.12	41.2	0.11	0.97

Supplementary Table 3C:

Targeted population	Cytokine	Vehicle control	CL075 (10 μM) + 2'3'-cGAMP(10 μM)	UM-3003 (10 μM)
CBMC	IFNγ	316.79	2919.42	1176.03
	IL-13	116.22	267.60	340.99
	IL-17A	2.83	1.54	60.57
	IL-2	43.75	65.58	30.5
	IL-4	257.43	930.54	874.9

Supplementary Table 3D:

Targeted population	Cytokine	Fold-change of CL075 + 2'3'-cGAMP over vehicle control	P-value (CL075 + 2'3'-cGAMP vs. vehicle control)	Fold-change of UM-3003 over vehicle control	P-value (UM-3003 vs. vehicle control)	P-value (CL075 + 2'3'-cGAMP vs. UM-3003)
CBMC	IFNγ	9.22	0.002 (**)	3.71	0.07	0.23
	IL-13	2.30	0.36	2.93	0.11	0.83
	IL-17A	0.54	0.66	21.41	0.007 (*)	0.002 (**)
	IL-2	1.50	0.10	0.70	0.24	0.007 (*)
	IL-4	3.61	0.18	3.40	0.08	0.71
